# Genetic variation associated with infection and the environment in the accidental pathogen *Burkholderia pseudomallei*

**DOI:** 10.1038/s42003-019-0678-x

**Published:** 2019-11-22

**Authors:** Claire Chewapreecha, Alison E. Mather, Simon R. Harris, Martin Hunt, Matthew T. G. Holden, Chutima Chaichana, Vanaporn Wuthiekanun, Gordon Dougan, Nicholas P. J. Day, Direk Limmathurotsakul, Julian Parkhill, Sharon J. Peacock

**Affiliations:** 1 Mahidol-Oxford Tropical Medicine Research Unit, Faculty of Tropical Medicine, Mahidol University, Bangkok, 10400 Thailand; 2 Bioinformatics and Systems Biology Program, School of Bioresource and Technology, King Mongkut’s University of Technology Thonburi, Bangkok, 10150 Thailand; 3 Wellcome Sanger Institute, Hinxton, CB10 1SA UK; 4 Department of Medicine, University of Cambridge, Cambridge, CB2 0QQ UK; 5 Quadram Institute Bioscience, Norwich, NR4 7UQ UK; 6 Faculty of Medicine and Health Sciences, University of East Anglia, Norwich, NR4 7TJ UK; 7 School of Medicine, University of St Andrews, St. Andrews, KY16 9TF UK; 8 Department of Mathematics, Faculty of Science, King Mongkut’s University of Technology Thonburi, Bangkok, 10140 Thailand; 9 Centre for Tropical Medicine, Nuffield Department of Medicine, University of Oxford, Oxford, OX3 7LF UK; 10 Department of Veterinary Medicine, University of Cambridge, Cambridge, CB3 0ES UK

**Keywords:** Genome-wide association studies, Evolutionary genetics, Bacterial genetics

## Abstract

The environmental bacterium *Burkholderia pseudomallei* causes melioidosis, an important endemic human disease in tropical and sub-tropical countries. This bacterium occupies broad ecological niches including soil, contaminated water, single-cell microbes, plants and infection in a range of animal species. Here, we performed genome-wide association studies for genetic determinants of environmental and human adaptation using a combined dataset of 1,010 whole genome sequences of *B. pseudomallei* from Northeast Thailand and Australia, representing two major disease hotspots. With these data, we identified 47 genes from 26 distinct loci associated with clinical or environmental isolates from Thailand and replicated 12 genes in an independent Australian cohort. We next outlined the selective pressures on the genetic loci (dN/dS) and the frequency at which they had been gained or lost throughout their evolutionary history, reflecting the bacterial adaptability to a wide range of ecological niches. Finally, we highlighted loci likely implicated in human disease.

## Abstract (web summary)

Claire Chewapreecha et al. combine 753 newly sequenced Thai *Burkholderia pseudomallei* isolates with 258 Australian isolates to identify genes associated with either clinical or environmental strains. They find 47 genes, 12 of which replicate in both locations, that may provide clues to the strategy used by this microbe to adapt to survive in wide range of ecological niches, including human hosts.

## Introduction

*Burkholderia pseudomallei* is an environmental Gram-negative bacterium and the cause of melioidosis, a serious infectious disease. A recent modelling study predicted that an estimated 165,000 people were affected globally per year, 89,000 of which died^1^. The bacterium has a broad range of ecological niches, and can be isolated from soil, surface water, amoebae, plants and infected humans and other animals in many tropical and sub-tropical regions^2–4^. Human infection results from environmental exposure associated with inoculation, ingestion or inhalation of the bacterium, with increasing risk of acquisition for people with predisposing health conditions or activities that increase exposure to soil or water, such as rice farming or drinking untreated water^5^. Infection can be acute, chronic, latent or cleared^6^, with rare cases of human-to-human transmission being reported^7,8^. Antibody responses to *B. pseudomallei* can be found in healthy individuals living in endemic areas in the absence of clinical symptoms^9,10^, suggesting that the majority of the exposure is harmless or results in sub-clinical infection.

*B. pseudomallei* can be found in the stool of some infected humans^11^ and experimental murine models^12^. This provides a potential mechanism for human-to-environmental transmission and the possibility of repeated passage through the human host. Serial passage of *Burkholderia cenocepacia* in a long-term chronic airway infection model in mice has been shown to increase bacterial fitness^13^. Based on this observation, the natural passage of *B. pseudomallei* through humans, other animals or its natural predators such as soil amoebae might have enhanced and maintained selection pressure for pathogenicity in a subset of the population. This potentially results in heterogeneity of bacterial virulence, as evidenced by marked variations in severity and pathogenicity in mice challenged by different *B. pseudomallei* strains^14–16^. *B. pseudomallei* has a large and highly variable accessory genome across the species^17–19^. While the core genome may be sufficient for strain survival, it is possible that specific bacterial genes, gene variants or their combinations may confer additional advantages for survival and replication in specific niches including human infection, or particular environmental conditions. Here, we sought evidence for bacterial genetic factors associated with human disease and environmental adaptation using two independent datasets from major melioidosis hotspots in Thailand, and Australia^18–23^. These were used as a discovery and validation dataset, respectively.

## Results

### Clinical and environmental isolates are inter-mixed

**Fig. 1:**
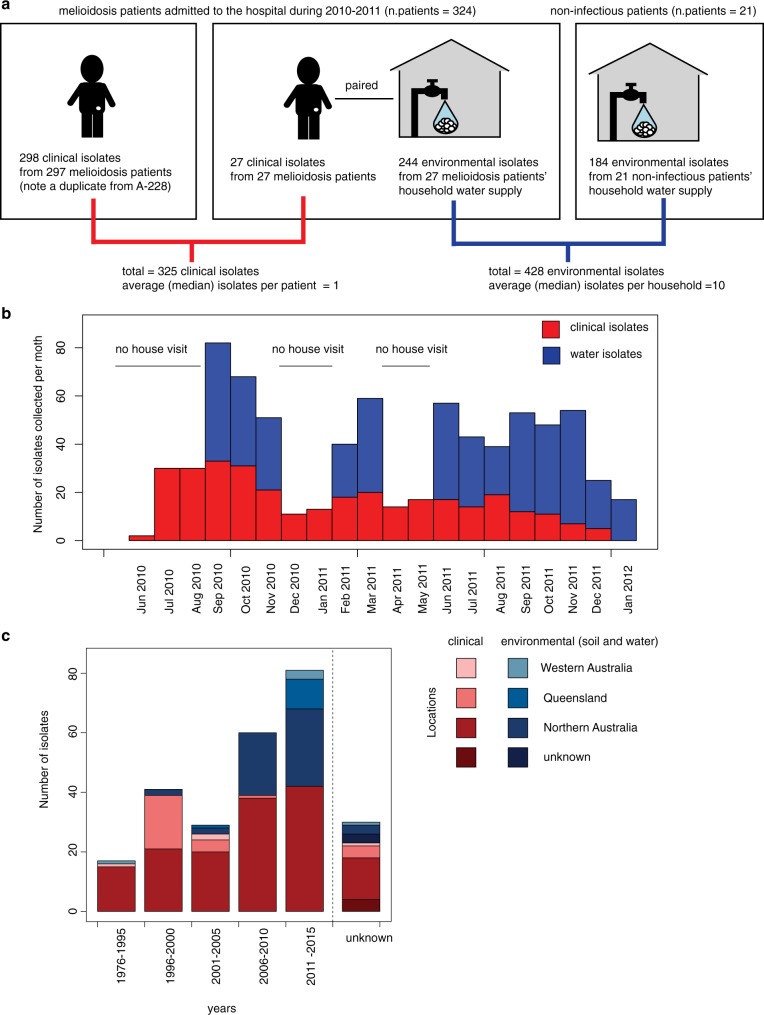
Sampling framework for *B. pseudomallei* isolates from the case control study. **a** The chart shows the number of clinical and environmental isolates from patients and/or household water supplies of cases (patients with melioidosis) and controls (patients with non-infectious conditions admitted during the same period). **b** Temporal distribution of environmental and disease isolates in the discovery dataset collected from June 2010 to January 2012. With the exception of months with no house visits, the number of monthly clinical and environmental samples collected were positively correlated (linear regression, adjusted *R*-square = 0.259, *p* value = 0.026). **c** Spatial and temporal distribution of environmental and disease isolates in the validation dataset from the public database.

**Fig. 2:**
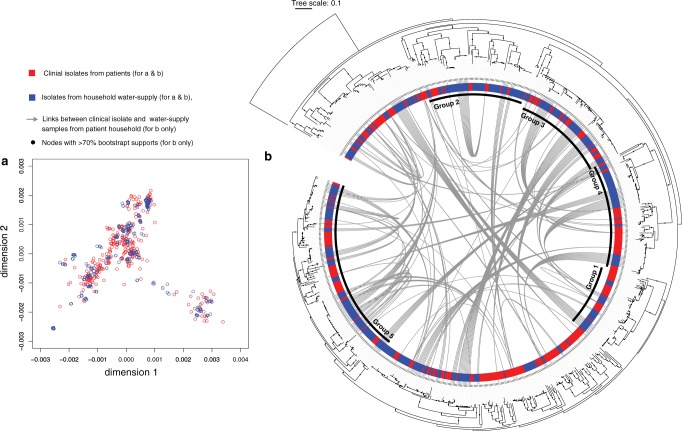
Population structure and phylogeny of *B. pseudomallei* isolated from patients and their household water supplies in northeast Thailand. **a** Multi-dimensional scaling based on the two dimensions that best explained data variability. **b** Maximum likelihood phylogeny generated from core gene SNPs in northeast Thailand isolates rooted on an Australasian outgroup Bp668. Nodes with bootstrap support of >70 are shown by black dots, with inner black rings presenting 5 monophyletic groups where detailed phylogeny-based analyses were performed. The outer coloured ring shows the isolate source. The grey arches represent an analysis of 27 cases who had a clinical isolates and up to 10 isolates cultured from their water supply, showing the connections for isolates from each case. Source data used to plot (**a**) is available in Supplementary Data 11.

We first outlined the population structure of the dataset from Northeast Thailand where information from household sampling structure was also available. *B. pseudomallei* used in this collection was originally cultured from patients presenting to Sunpasitthiprasong hospital in Ubon Ratchathani between 2010 and 2011, together with residential water sources from melioidosis patients as well as non-infected individuals^5^ (see Methods for details). With the exception of 1 patient where two isolates were cultured, a single isolate was collected from each patient (*n* patient = 324, *n* clinical isolates = 325). Up to 10 water isolates were sampled from each household (*n* households = 48, *n* environmental isolates = 428, see Fig. 1 for sampling framework). Unlike many pathogens where isolates associated with disease contain substantially fewer genes^24,25^, a pan-genome analysis revealed a similar number of genes per genome in clinical and environmental isolates (two-sided Mann–Whitney *U* test, *p* value = 0.312). Moreover, both phylogenetic and multidimensional scaling approaches (MDS) indicated that clinical and environmental isolates were largely mixed with each phylogenetic group comprising both clinical and environmental isolates (Fig. 2).

Previous studies have noted the importance of recombination in driving *B. pseudomallei* evolution^23,26–28^, demonstrating genetic interactions and co-evolution of multiple *B. pseudomallei* lineages that shared the same habitat. Evidence for genetic interactions^29^ between clinical and environmental isolates was sought for 5 monophyletic groups, each of which had ≥70% bootstrap node support on the core genome phylogeny to ensure robust analysis (Supplementary Fig. 1). Our results showed that both clinical and environmental isolates in each group had undergone recombination. Moreover, similar numbers of recent recombination events (defined by recombination located at the tips of the phylogeny) were identified in both clinical and environmental isolates (Fisher’s exact test *p* value = 1, Supplementary Fig. 2). A search for the sources and sinks of recent recombination events (see Methods) revealed that clinical isolates could act as DNA donors for recombination detected in environmental isolates. Similarly, environmental isolates could act as DNA donors for recombination detected in clinical isolates. DNA recipients and DNA donors were more likely to be found in isolates from the same origin. There was a higher probability of clinical isolates being the donor for clinical isolate recipients (two-sided Mann–Whitney *U* test *p* value < 2.2 × 10^−16^), and environmental isolates being the donor for environmental isolate recipients (two-sided Mann–Whitney *U* test *p* value = 9.41 × 10^−9^). Together, this suggests a structure to the genetic flux within the clinical and environmental isolates despite the potential for ecological mixing of the population.

### Not all environmental exposure leads to infection

**Fig. 3:**
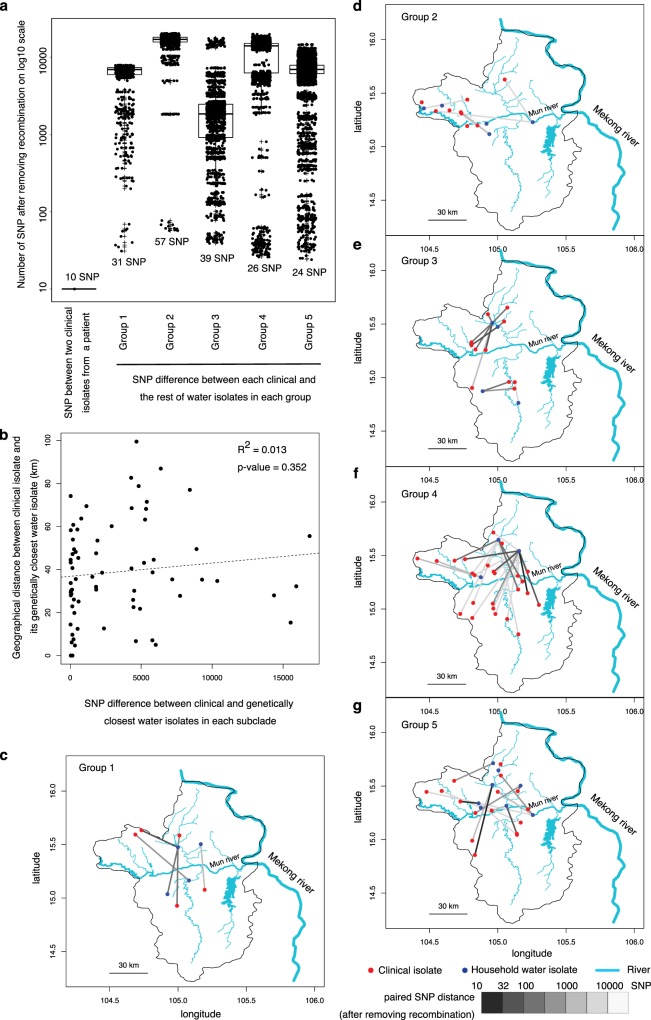
Genetic relatedness between clinical and environmental isolates from households. **a** Boxplot summarises pairwise SNPs distance between each clinical and its closest environmental isolate from each monophyletic group after removing recombination signals. A pairwise SNP distance between two clinical isolates cultured from the same patient were included as a threshold. **b** Correlation between pairwise SNP distance and geographical distance of clinical and its closest environmental isolates. **c**–**g** Geographical distance between clinical and its closest environmental isolates by monophyletic group. Red and blue dots represent clinical and environmental isolates, respectively. Colour shade of the links indicates the pairwise SNP distance between the pair. Source data used to plot (**a**) and (**b**) is available in Supplementary Datas 12 and 13, respectively.

We next investigated the potential source of infection by comparing the genetically closest environmental isolates to each clinical isolate using the Northeast Thailand data. Given that consumption of untreated household water supply was common in this endemic area^5^, we first considered the link between household water supply and infection. Of 48 households with water samples cultured positive for *B. pseudomallei*, 27 households belonged to melioidosis patients. Notably, only 6 households of melioidosis patients had environmental and clinical isolates clustered within the same monophyletic group (Fig. 2b, Supplementary Fig. 3a). After removing signals from recombination, comparison of pairwise genetic difference showed that clinical and environmental isolates from these 6 households (median = 6994 single-nucleotide polymorphism (SNP)) were not significantly more similar to one another than to those from randomly paired clinical and environmental isolates (median = 7,090, Mann–Whitney test *p* value = 0.3901, Supplementary Fig. 3b). This result indicated that the studied patients did not commonly contract melioidosis from their household water supply. It is possible that the infecting isolate represented a minority population in water that was not detected in the study, or it was acquired elsewhere. The availability of Global Positioning information for 134 clinical and 387 environmental isolates allowed us to locate the potential source of infection for a subset of melioidosis cases. After removing signals from recombination events, we mapped the pairwise genetic differences between each clinical isolate and its closet environmental isolate (range: 24–16,866 SNPs, Fig. 3a) and their geographical distance (range: 5–100 km apart, Fig. 3b). We found a lack of genetic and spatial correlation between clinical isolates and their closest environmental isolate (*R*^2^ = 0.013, *p* value = 0.352) with no genetic evidence that patients had acquired *B. pseudomallei* from their neighbourhood or farmland (defined as 10 km^2^ from patient’s household). It is possible that the Mun river, its extensive canal systems and floodplains^30^ may have dispersed genetically close isolates over a large geographical distances (Fig. 3c–g), thereby disrupting the genetic and spatial correlation. It is also likely that our environmental isolates were not sufficiently intensively sampled to capture the source of infection. Nevertheless, the lack of conclusive cases of household contraction despite evidence of exposure supports the hypothesis that not all *B. pseudomallei* exposure leads to infection.

### Genetic factors associated with disease and the environment

We next investigated potential genetic signals that were associated with infection or the environment by estimating the correlation between the bacterial phylogeny and distribution of source of isolation on the tree using Pagel’s *λ*^31^. Only five monophyletic groups were included in the tests to ensure robust analysis. The distribution of “disease” and “environmental” origins was not random (Supplementary Fig. 4), indicating that there may be separable environmental and clinical clades either at deep or shallow nodes^32^. This could reflect the presence of bacterial determinants that mediate survival in human or environmental niches.

**Fig. 4:**
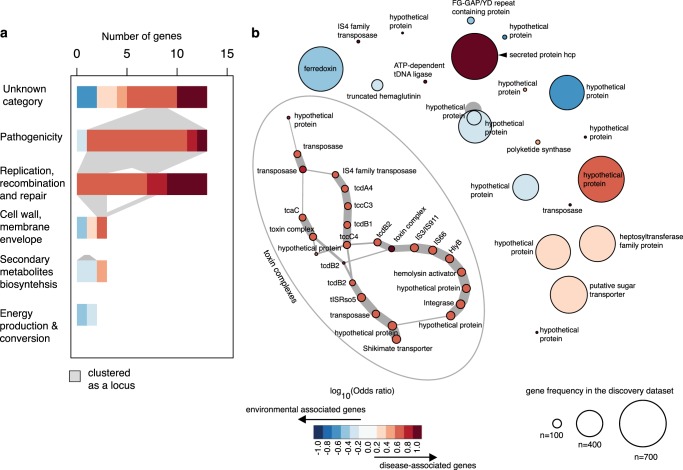
*B. pseudomallei* disease- and environmental-associated genes. **a** Bar charts summarise the frequency of disease- or environment- associated genes by functional category. The plots are ranked by categorical gene frequency from unknown category (*n* = 13 genes), potential roles in pathogenicity (*n* = 13 genes), replication, recombination and repair (*n* = 13 genes), cell wall membrane envelope biogenesis (*n* = 3 genes), secondary metabolite biosynthesis (*n* = 3 genes), and energy production and conservation (*n* = 2 genes). **b** Distance network reveals genetic loci enriched in disease- and environment-associated isolates. A network was constructed on distance between disease and environmental-associated genes that fell within the size of operon described by the transcriptional unit, as reported in Ooi et al. 2013. Each node represents each gene, with the edge thickness proportional to the frequency of each gene pair observed in the population. The largest disease-associated locus identified in this dataset was the toxin complex. For **a** and **b**, the colour indicates the effect size and directionality of association on the scale of log_10_(Odds ratio), with red and blue presenting association with disease and the environment, respectively.

We applied two complementary genome-wide association studies (GWAS) (a kmer-based^33^ and a pan-genome based^34^ approach) to the 325 clinical and 428 environmental isolates, which were controlled for population stratification (see Methods, Supplementary Datas 1 and 2). We note that there was potential cross categorisation as the environmental isolates could be capable of causing disease. While this caveat reduces the power to detect the association which elevates the true negatives, this would be unlikely to impact on the false-positive rate. Of 24,856,071 kmers used to define the population, 38,797 (0.156%) were associated with “disease” or “environmental” origin. These were mapped onto the pan-genome to identify potential genes, resulting in 365 “disease-associated” or “environmental-associated” genes. The pan-genome based GWAS analysis identified 675 disease-associated or environment-associated genes. Comparison of output from the two methods showed that 47 genes were detected by both (38 disease-associated and 9 environmental-associated genes, Supplementary Datas 3 and 4), which account for 0.3% of the pan-genome. Based on the size of transcriptional operons reported in Ooi et al.^35^, we grouped these genes into 26 loci (Fig. 4). These 47 genes were evaluated in an independent dataset from Australia (clinical isolates = 184, environmental isolates = 73), which showed that 12 genes (25.5%) were either enriched in clinical or environmental isolates (Supplementary Data 5, two-sided Fisher’s exact test, FDR < 0.01). The fact that isolates from Australia and Southeast Asia represent distinct phylogenetic clades^19,23,28^ is consistent with parallel evolution for a proportion of the disease-associated and environment-associated genes.

Functional enrichment analyses of the 47 gene clusters in the discovery cohort showed an elevated frequency of the term “Pathogenesis” and “Replication, recombination and repair” (Supplementary Data 6, one-sided Fisher’s exact test *p* value 2.30 × 10^−7^, and 2.08 × 10^−12^, respectively). The former may allow the bacterium to compete in specific environmental niches or survive inside single-cell or multicellular organisms during infection. Genes annotated with the term “Replication, recombination and repair” largely comprised transposons that may act as markers or remnant elements for horizontally transferred genes, or may inactivate gene function. Apart from these, 8 of 26 loci consisted of IS, transposons and integrase, which highlights the significance of transposable elements in rearranging bacterial genomes.

### Selection pressures maintaining niche-associated genes

**Fig. 5:**
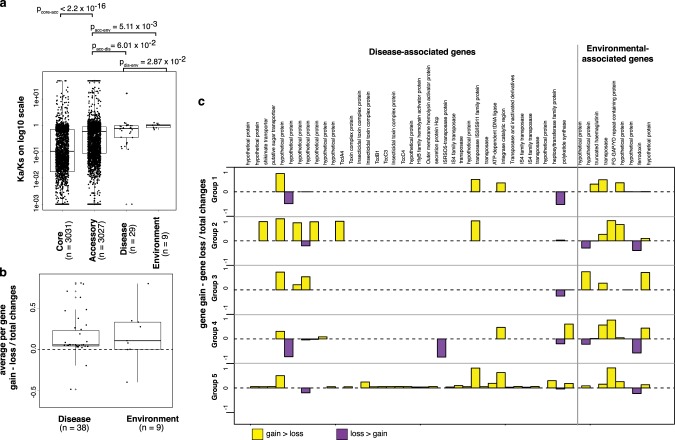
The selective pressure on disease- and environmental associated genes and the frequency at which they had been gained or lost throughout their evolutionary history. **a** The dN/dS of core genes, accessory genes, disease-associated genes, and environmental-associated genes are plotted on a log 10 scale. Two-sided Mann–Whitney *U* test was used to compare categorical observation. **b** The ratio of gene gain minus gene loss over the total gain and loss events for disease-associated genes and environmental associated genes. Independent observations were drawn from five monophyletic groups. ANOVA was employed to test the differences in group observation, where available treated as replicates for each gene. Where multiple observations were observed for each gene, a mean across different monophyletic groups was taken as an average. For **a** and **b**, boxplots summarise the distribution of data based on first quantile, median and third quantile. **c** A summary of net gain or loss events across all five groups. Yellow and purple bars indicate greater net gain and greater net loss of each gene. Source data used to plot (**a**) and (**b**) is available in Supplementary Datas 14 and 15, respectively.

We explored whether or not the 38 disease-associated and 9 environmental-associated genes were under selective pressure by calculating the ratio of the rate of non-synonymous substitutions per non-synonymous site to the rate of synonymous substitutions per synonymous site (dN/dS). The average for both groups was below 1, but the ratio was significantly higher for environmental-associated compared with disease-associated genes and accessory genes (Fig. 5a, Supplementary Data 3, Mann–Whitney *U* test *p* value = 2.87 × 10^−2^ and 5.11 × 10^−3^, respectively). Despite the small number of genes being compared, this suggests that the subset of genes in the environment-associated genes may be under reduced purifying selection, or elevated diversifying selection, compared to disease-associated and other accessory genes. We further quantified the number of times each cluster was acquired or lost in monophyletic groups that constitute an entire phylogenetic tree (*n* group = 5, *n* of isolate in each group ≥57 isolates, node bootstrap supports ≥70). Assuming an equal rate of gene gain and loss, stochastic mapping of the presence of each disease- or environment-associated cluster highlighted multiple gene gain-and-loss events, one possible reason for which is a constant change in niches that may include switching between extra- and intracellular lifestyles. Notably, 38/47 genes showed a preference for net gain, 4/47 had a preference for net loss, while 5/47 showed ambiguous directions when compared across multiple monophyletic groups (Fig. 5b, c, Supplementary Data 3). Although we did not observe differences in net gain or loss between disease- and environmental-associated genes (ANOVA test, gene *p* value = 0.841, loci *p* value = 0.876), our results highlighted a greater proportion of overall net gain for both disease- and environmental associated genes. Some of these may confer the bacterium longer-term advantages, which warrants further investigation.

### Examples of disease-and environmental associated genes

Many of the disease-associated loci contained genes that encoded biologically plausible or known virulence determinants. One example was a large toxin complex (*tcdB*, *tcdA*, *tccC* and hemolysin activator *fhaC*) encoded by a locus of up to 69.7 kb, which was identified in the discovery dataset (Supplementary Fig. 5). This locus has not been characterised in *B. pseudomallei* but homologues exist in diverse bacterial species including *Pseudomonas*, *Yersinia* and *Photorhabdus*^36,37^. The latter is an insect pathogen, experimental characterisation of which has demonstrated that *tccC* has enzymatic activity^38^ and that *tcdA* and *tcdB* facilitate the translocation of the toxin into host cells^37^. These toxin genes were flanked in *B. pseudomallei* by several integrases and transposases families including IS*2*, IS*3*/IS*911*, IS*4*, IS*66*, IS*166*, IS*407*, IS*111A*/IS*1328*/IS*1533* and IS*1478*, indicative of a mobile genetic element origin. An analysis of gene gain-and-loss events for the locus was possible for one monophyletic group (group 5, *n* = 156 isolates), as this locus was variably present in group 5 but fully present or absent in the other groups. For this group, we observed a slightly greater net gain of the whole locus with the toxin genes being acquired and lost 10 and 9 times, respectively. This may suggest not only a selective advantage but also a fitness cost associated with this locus for *B. pseudomallei*.

An example of environmental-associated loci is a truncated variant of filamentous hemagglutinin (*fha*), a known adhesin and immunomodulator across different bacterial species. In *B. pseudomallei*, the number of *fha* genes varies between isolates and different combinations of *fha* genes have been observed with patients infected by *B. pseudomallei*, with a specific *fha* variant reported to have increased risk of infection associated with positive blood cultures^39^. While our kmer approach identified disease-associated signals from haemaglutination activity domains on this gene, our pan-genome approach detected environmental-associated signals from a truncated form of this gene (Supplementary Fig. 6). A closer inspection highlighted a truncation caused by a premature stop codon upstream of the haemaglutinin repeat domains, which might disrupt gene function. This environmental-associated and truncated form showed a greater net gain in all tested monophyletic group (Fig. 5), suggesting a selective advantage of this variant in the northeast Thailand setting.

## Discussion

Our results suggest that despite evidence of direct contact with householders, not all *B. pseudomallei* exposure led to infection. A transition from exposure to disease likely requires additional risk factors involving *B. pseudomallei*, host and environment. Our analyses have identified *B. pseudomallei* gene clusters that are enriched in clinical or environmental isolates. These genes have arisen repeatedly in different populations with distinct phylogeography, demonstrating robustness of the findings from the Thai discovery dataset. Many of these genes are under relaxed purifying selection and have been gained or lost multiple times throughout the organism’s evolutionary history, implying that there may be several niches to which this opportunistic bacterium is adapted. This includes environmental and other eukaryotic hosts^3,40,41^, the latter potentially providing genetic pre-adaptation for invasion and survival in the human host. Based on our current knowledge of the ecology of *B. pseudomallei*, there are still a substantial number of disease-associated and environment-associated genes with unknown function, unidentified interaction partners or unexplored roles in each ecological niche, thereby limiting the immediate translational applications of our study. Further exploration into the ecological role of these genes will be essential to better manage and prevent the infection from this accidental pathogen.

## Methods

### Bacterial isolates

Two bacterial collections were used to create independent discovery and validation datasets. These originated from distinct regions where melioidosis is highly endemic—northeast Thailand and northern Australia^18–22^.

The discovery dataset was drawn from a study of the activities of daily living associated with melioidosis, which was conducted at Sunpasitthiprasong (formerly Sappasithiprasong) hospital in Ubon Ratchathani, Northeast Thailand between 2010 and 2011^5^. In brief, 330 cases of culture-proven melioidosis and 513 control patients with non-infectious conditions were recruited^5^. *B. pseudomallei* can survive in water^42^, an ability contributing to its environmental persistence in the endemic area. Five litres of residential drinking water were collected per household and cultured for *B. pseudomallei* from cases and controls who lived within 100 km of the hospital. *B. pseudomallei* was isolated from 12% of borehole and tap water samples, and 4% of well water samples. Multiple colonies were picked and individually saved from each water sample. Consumption of untreated water was common (85% of cases and 72% of controls) and associated with a higher risk of melioidosis^5^. We assumed that isolates from water were a fair representation of environmental isolates. Simultaneous infection with more than one strain of *B. pseudomallei* was reported to be uncommon^43^. Except for 1 case, a single colony was cultured from each melioidosis patient. We noted a differential rate of *B. pseudomallei* being cultured from clinical (median for blood culture = 1 CFU/mL)^44^ and water samples (median = 1 × 10^−3^ CFU/mL)^5^. For the purposes of the study described here, we sequenced 325 *B. pseudomallei* isolates from 324 cases, and 428 *B. pseudomallei* colonies (isolates) from 48 water samples (including samples from 27 melioidosis patients) (Fig. 1, Supplementary Data 1).

The validation dataset consisted of whole genome sequence data for 258 B. pseudomallei isolated in Australia, which were downloaded from the NCBI database (Supplementary Data 2). These isolates have been described previously^18–23^. In brief, isolates were from patients with melioidosis (*n* = 184) and the environment (*n* = 73). The temporal and spatial distribution of isolates in this dataset is summarised in Fig. 1.

### Whole-genome sequencing

DNA was extracted from the 753 Thai *B. pseudomallei* isolates as described in^45^. DNA libraries were prepared according to the Illumina protocol and sequenced on an Illumina HiSeq2000 with 100-cycle paired-end runs giving a mean coverage of 84 reads per nucleotide. Sequencing of clinical and environmental isolates was done at the same time on the same platform. Taxonomic identity was assigned using Kraken^46^ to control for potential contamination in each sample with other closely related species. While the data generated should represent two chromosomes, the plasmid is frequently lost during culture and was lacking from many of the short read data sets.

### Genome assembly and pan-genome analysis

New assemblies were performed as described in ref. ^47^ to give a median of 97 contigs (min = 61 contigs, max = 259 contigs), and median length of 7,114,540 bp (min = 6,884,381 bp, max = 7,404,549 bp). All study genomes were annotated using Prokka^48^. A predicted median of 5936 coding sequences were assigned onto each genome (min = 5762, max = 6264), which falls within the range of published reference genomes^40,49^. Roary^50^ was used to calculate the pan-genome for the discovery dataset together with the two reference *B. pseudomallei* genomes (K96243 from Thailand and Bp668 from Australia). The inclusion of the well-characterised Thai reference K96243 served as the quality control for the pan-genome analysis, and the Australian reference Bp668 served as an outgroup to root the phylogeny in a subsequent analysis. An all-against-all BLASTP comparison at 92% sequence identity was used as described in ref. ^19^. Genes were defined as core if present in ≥99% of isolates. This led to 4322 and 10,718 genes being classified as core and accessory, respectively (Supplementary Data 7). The number of core genes identified fell within the range described previously^18^.

### Population structure estimated by multi-dimensional scaling

The population structure of the 753 Thai isolates was estimated from sequence assemblies using Mash v. 1.1.1^51^, which captures information from intergenic regions, core and accessory genes. Assemblies were shredded into their constituent kmers. The pairwise distance between assemblies was estimated and computed into the 753 × 753 matrix. Metric MDS was performed using R cmdscale to project the population structure into n-1 coordinates. The top three coordinates were used to control for GWAS population stratification.

### Population structure estimated by phylogenetic trees

Phylogenetic approach was employed to determine the overall population structure as well as more detailed subclade analyses. An overall population structure was estimated using SNPs in the core genome. Single-copy core genes from 753 isolates, K96243 and Bp668 were concatenated and aligned using Mafft v7.205^52^, followed by manual inspection using SeaView^53^. This comprised 4322 genes, representing on average 73% of genes in individual genomes. Single-nucleotide substitutions in the alignment were called using the methods described by Page et al.^54^, resulting in SNPs. A maximum-likelihood phylogeny was constructed with RAxML HPC v.8.2.8^55^ using a general time reversible nucleotide substitution model with four gamma categories for rate heterogeneity and 100 bootstrap support. The overall phylogeny had 58.4% of external and internal nodes showing ≥70% bootstrap support.

For measuring phylogenetic signals and ancestral reconstruction analyses, only monophyletic branches with >70% bootstrap support were considered. Branches with poor bootstraps were removed using iTOL^56^. Subsequent tests were performed on 5 monophyletic groups comprising group 1 (*n* = 57), group 2 (*n* = 84), group 3 (*n* = 86), group 4 (*n* = 91) and group 5 (*n* = 156), totalling 474 isolates (63% of the northeast Thailand dataset). Each group was rooted on the Australia isolate Bp668.

Maximum-likelihood phylogenies were also used to examine specific disease-associated clusters by concatenating and aligning genes using Mafft v7.205^52^, with truncated genes manually checked. A maximum-likelihood phylogeny was constructed as above with 100 bootstrap support and rooted on an Australian gene homologue.

### Detection of recombinant sites

Recombination detection required genome alignment with higher resolution. A pseudo-alignment from each group was generated by mapping sequence reads against a reference genome K96243^49^ from northeast Thailand. Methods described in ref. ^57^ was applied to allow greater sensitivity for detection of variants including small insertions and deletions (indels). To determine the impact of recombination within this dataset, we ran Gubbins^29^ on individual monophyletic group (Supplementary Fig. 1). The regions identified as recombinogenic were largely those reported as genomic islands^58^. The contribution of recombination to the overall diversity was estimated by ratio of recombination events to the number of mutations (r/m) thus avoiding a bias introduced by using number of SNPs that can be affected by DNA donors of varying genetic distances. Phylogenies with recombination removed were used to determine connections between isolates from the clinic and household water supply.

### Identification of recombination donors

Potential sources of recombination fragments were determined by comparing the sequences identity to the recombined fragments detected the recipient strains. Recombination regions overlapped with genomic islands mobile genetic elements were excluded. Identification of potential recombination donor were focused on recent recombination events with recipient located on the tip of each subclade phylogeny. Recipient blocks were searched using BLAT v.35^59^ against the rest of the assemblies for identical match (donor blocks). To minimise non-specific match, the search was restricted to recipient block >10 bp with no unknown nucleotide “N” detected in both recipient and donor blocks.

We next calculated the probability of each isolate being a donor for individual recipient isolate. For each recipient isolate where “*n*” potential donor isolates were identified, each potential donor isolate was assigned a probability of “1/*n*”. Isolates showing no hit for a particular search were assigned probability of 0. The total likelihood of each isolate for being a donor was calculated as the sum of the above probabilities from all donation events.

### Mapping geographical distance

Information of the Global Positioning System were available for 521 isolates (Supplementary Data 1). The pair-wised distance between isolates were calculated using R package geosphere^60^ with Haversine function.

### Estimation of phylogenetic signals

Pagel’s *λ*^31,61^ was used to assess phylogenetic signal in each monophyletic group, where bootstrap supports ≥70%. This quantitative measurement helped determine whether members of the same group were more similar than those outside of the group, and whether the search for genetic signals that distinguish the two groups was productive. Origin of isolation (clinical or environmental) was reconstructed onto the tree using fitDiscrete from the R package Geiger^62^. We compared the model fit of the tree using log-likelihood of the untransformed maximum likelihood tree against the model where the tree was transformed to a polytomy or partially transformed trees (internal branches were multiplied by *λ* = 0, 0.1, 0.2, 0.3, 0.4, 0.5, 0.6, 0.7, 0.8, and 0.9, Supplementary Fig. 4a). We also reconstructed randomised origin of isolation (clinical or environmental, 100 permutations) onto the tree and compared log-likelihood scores obtained from reconstruction with the actual origin versus randomised origins.

### Detecting kmers associated with disease and the environment

Two separate GWAS were performed to screen kmers for associations with source of isolation (clinical or environmental) using the 753 Thai genomes. Assemblies were shredded into overlapping kmers of 9–100 bases, resulting in 24,856,071 kmers. All kmers occurring in more than one assembly were counted using fsm-lite (https://github.com/nvalimak/fsm-lite) as described in ref. ^33^, and filtered to retain kmers that appeared in 5–95% of samples (“fsm-lite –v –s 5 –S 95 –l lists.txt –t index > kmer”; followed by “gzip kmer”). Kmers with low frequency (5% minor allele frequency cut-off) were removed and thus reduced the data to 24,555,746 kmers. Kmers were next filtered using the *χ*^2^-test (1 d.f.). Kmer association with a *p* value < 10^−5^ were has been shown previously through simulations to be true positive associations^33^, and thus was retained for further investigation. This step reduced the kmers to 300,325 kmers. Seer^33^ was then used to fit a logistic curve to binary data (clinical or environmental) for each kmer (“seer–pheno clin.env.pheno.tsv -k fsm_kmer.{i}.gz –struct structure.tsv –threads 4 > significant_kmers.{i}.txt”, where {i} is the job array). The first three principal components calculated from metric dimensional scaling were used as covariates to control for bacterial population structure. This resulted in 37,104 kmers positively associated with clinical isolates, and 1693 kmers positively associated with environmental isolates (Supplementary Data 8). All kmers were mapped to the K96243 reference^49^ and the raw assemblies of 753 isolates using BLAT v. 35^59^ to identify the relevant genes and gene variants. In order to map low complexity kmers (length 10–26 base pairs), the following parameters were used: blat –minMatch = 1 –tileSize = 8 –minScore = 10. The match was allowed for both forward and reverse strands. Only identical hits were retained. Any predicted coding sequences (CDS) with more than 2 kmer hits were pooled and collectively termed “disease-associated” genes or “environment-associated” genes. These kmers were shown to represent both small-scale (single polymorphic and indels) and large-scale (likely introduced via horizontal gene transfer) variation^19^.

### Detecting genes associated with disease and the environment

As a complementary approach, we performed a pan-genome based GWAS using Scoary^34^ on the Thai dataset (Supplementary Data 9). Two separate GWAS were performed to find genes associated with source of isolation (clinical or environmental) while correcting for population structure using the phylogenetic tree (scoary –t clin.env.pheno.tsv –g gene_presence_absence.tsv –n tree –c BH). False-discovery rate (FDR) was estimated by Benjamini–Hochberg adjusted *p* value (with –c BH) provided in Scoary^34^, and tested for consistency against an empirical *p* value generated by random permutations (Supplementary Fig. 4b). Disease-associated or environment-associated genes with a Benjamini–Hochberg adjusted *p* value < 0.01 were reported and compared for consistency with genes identified by the kmer-based GWAS (Supplementary Data 3). Sequences of disease-associated and environment-associated genes were outlined in Supplementary Data 10.

### Validating genes associated with disease and the environment

Disease-associated or environment-associated genes that were identified by both the kmer-based and pan-genome based methods (*n* = 47) were validated in an independent dataset from Australia. Where genome assemblies were available, genes were validated by searching for Australian orthologues using BLAT v. 35^59^ allowing for 92% identity, the same cut-off used in the pan-genome analysis. Where short reads were available^23^, ARIBA^63^ was employed to perform local assembly and mapping to check for the presence or absence of genes. The sequence identity threshold (–cdhit_min_id 92) was adjusted to 92% for consistency. Gene distribution across Australian clinical and environmental isolates was tested using two-sided Fisher’s exact test with a Benjamini–Hochberg adjusted *p* value (Supplementary Data 5).

### Simulation on association analysis

As a complementary to FDR determined by Benjamini–Hochberg approach^64^; for each tested gene in both discovery and validation datasets, we separately ran 100 permutations with true genotypes (gene presence or absence) but randomised source of isolation (clinical or environmental). This generated an empirical *p* value to determine the cut-off threshold. For the discovery cohort, all candidate genes achieved significant association at an empirical *p* value < 0.01, suggesting that the observed associations were not random (Supplementary Fig. 4b). For the validation cohort, genes that could be replicated also achieved significant association at an empirical *p* value < 0.01. This also validated a Benjamini–Hochberg adjusted cut-off at *p* value 0.01 as our conservative threshold.

### Gene functional category

Gene ontology (GO) describing biological process, molecular function and cellular compartment was assigned to each gene in the pan-genome using InterProscan v5.21–60.0^65^. Not all genes matched the GO database. As of October 2019, 38.2% genes had GO terms assigned. A given gene could be associated with multiple GO terms (mean ~2.85, min = 1, max = 14), and when this occurred a parent GO term was used to represent child GO terms. Comparison of GO terms in disease versus environmental isolates, and their enrichment among disease-associated clusters versus expectation based on the reference genome K96243 was performed using one-sided Fisher’s exact test with all GO terms, with a Benjamini–Hochberg adjusted *p* value (Supplementary Data 6).

Disease-associated clusters were also annotated with Orthologous Groups of Proteins (COG^66^) and pathway maps (KEGG^67^ and MetaCyc^68^) to determine putative function. As of October 2019, COG, KEGG and MetaCyc could be assigned to 78.04, 9.79 and 7.72% of disease-associated genes, respectively. Information on protein domains was sourced from the Conserved Domain Database (CDD)^69^.

### Measuring gene selection pressure

The ratio of non-synonymous to synonymous substitutions (dN/dS or Ka/Ks) was calculated using the KaKs calculator^70^. To reduce computational load, we randomly selected accessory genes to represent equal number as core genes (*n* = 4322). Alignments of core, accessory, disease-associated and environment-associated genes were extracted from the pan-genome^50^. The test rejected neutrality (H_0_ dN/dS = 1, Fisher’s exact test *p* value < 0.05) in 3031 core genes, 3027 accessory genes, 28 disease-associated and 9 environment-associated genes. A non-parametric Mann–Whitney *U* test was used to investigate any departures in the mean of dN/dS for genes associated with disease, the environment and core.

### Estimating gene gain and loss events

Gain or loss of disease-associated and environment-associated genes through evolutionary history was quantified using make.simmap from the R package Phytools v 0.6–44^71^ with 1000 simulations. The analysis was performed separately for each monophyletic group. We first compared likelihood scores for the presence or absence of each gene across the phylogeny with the three different models (AR, ER and SYM). ER was the best fit model in our dataset and was selected. For each gene, only monophyletic groups with gene frequency between 0.01 and 0.99 were included in the analyses.

### Data visualisation

Visualisation of phylogenetic trees and statistical analyses was performed in R, Phandango^72^, and FigTree v 1.4.2 (http://tree.bio.ed.ac.uk/software/figtree/).

### Statistics and reproducibility

We employed chi-squared tests or Fisher’s exact tests to compare categorical variables, and parametric ANOVA or non-parametric Mann–Whitney *U* tests to evaluate continuous variables, respectively. Unless otherwise stated, two-sided tests were performed in all cases. Where appropriate, we used the Benjamini–Hochberg procedure and Monte Carlo permutation test to adjust *p* values for multiple comparisons, thereby controlling for multiple hypothesis testing. To ensure reproducibility, we also used two independent approaches to perform GWAS (kmers-based and gene-based methods) on the discovery dataset and validated the enrichment of candidate genes in an independent validation cohort. Source data used to plot Figs. 2a, 3a, b and 5a, b are archived in Supplementary Datas 11–15, respectively.

### Reporting summary

Further information on research design is available in the Nature Research Reporting Summary linked to this article.

## Supplementary information

Supplementary Information

Description of Additional Supplementary Files

Supplementary Data 1

Supplementary Data 2

Supplementary Data 3

Supplementary Data 4

Supplementary Data 5

Supplementary Data 6

Supplementary Data 7

Supplementary Data 8

Supplementary Data 9

Supplementary Data 10

Supplementary Data 11

Supplementary Data 12

Supplementary Data 13

Supplementary Data 14

Supplementary Data 15

Reporting Summary

Peer Review File

## Data Availability

All supporting data are included in this published article and its supplementary material. Short reads and assemblies for isolates are archived in ENA or NCBI database. Accession number for each individual isolate in discovery and validation dataset are given in Supplementary Datas 1 and 2. Source data for the figures are available in Supplementary Datas 11–15. Pan-genome analysis listing all genes in the dataset is available via Figshare^73^ (details in Supplementary Data 7) Sequences of disease- and environmental associated genes are available via Figshare^74^ (details in Supplementary Data 10).

## Code availability

All tools and R packages used for the analysis are publicly available and fully described in the method sections and noted in refs. ^33,34,46–48,50–56,59–63,70–72^.
