# Supplementary material for: Genetic variation associated with infection and the environment in the accidental pathogen Burkholderia pseudomallei
Source: Commun Biol. 2019 Nov 22;2:428. doi: 10.1038/s42003-019-0678-x (PMC6874650; doi:10.1038/s42003-019-0678-x)
Supplement: Supplementary file 1 — Supplementary Information [file 42003_2019_678_MOESM1_ESM.pdf]

Supplementary Information

Supplementary Figure 1

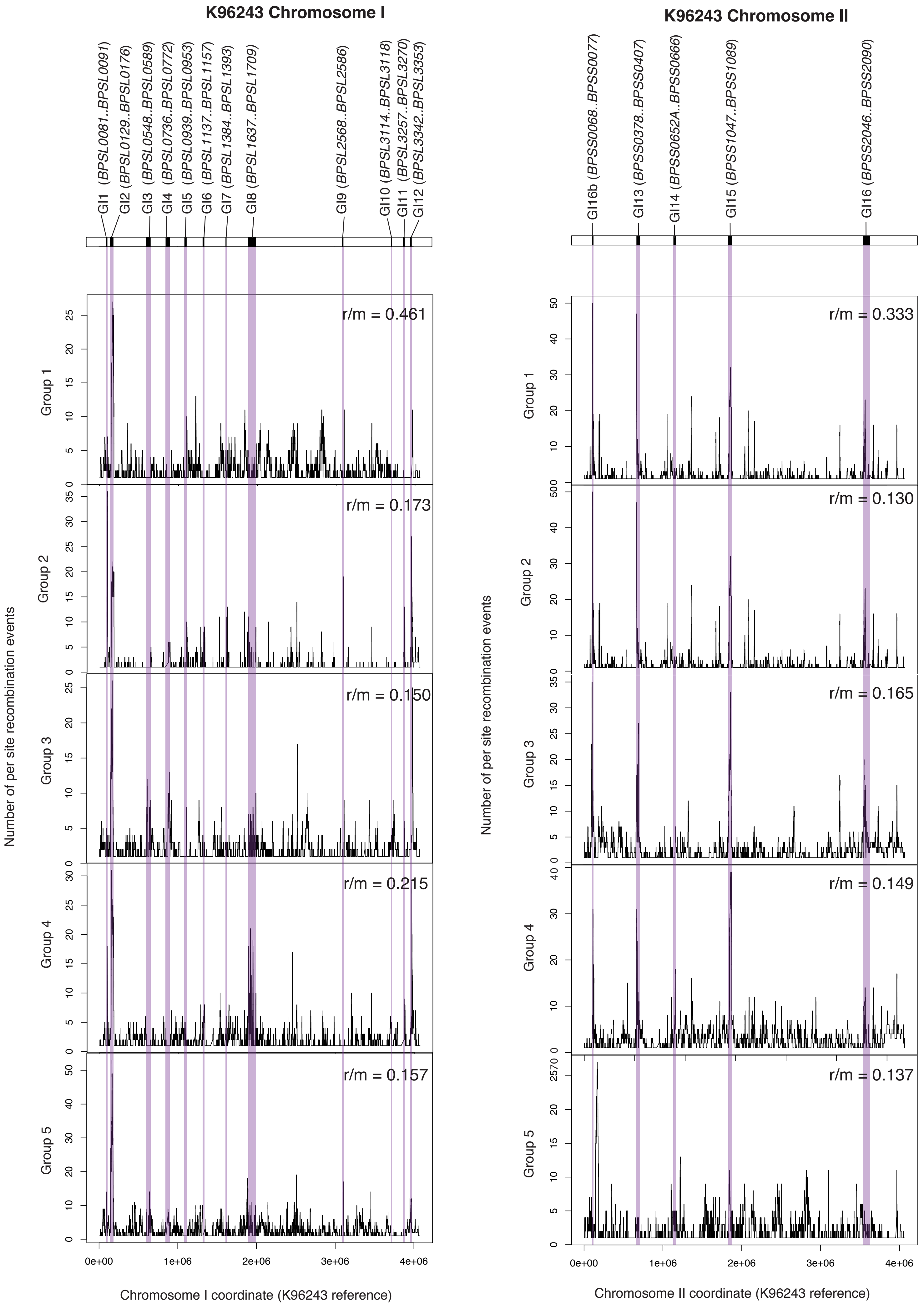

Supplementary Figure 1 Recombination hotspots coincide with genomic islands (GI). Panels (top to bottom) represent recombination detected in 5 monophyletic groups with columns highlighting recombination pattern in chromosome I (left) and II (right). Number of recombination events observed at each site were plotted on the vertical axis, revealing recombination hotpots. Shaded in purple are GIs previously characterized in K96243 strains, many of which coincide with recombination hotpots. Recombination events per mutation (r/m) across investigated groups were quantified by median of r/m on each branch.

Supplementary Figure 2

a Number of recent recombination events detected at tips of each monophyletic group

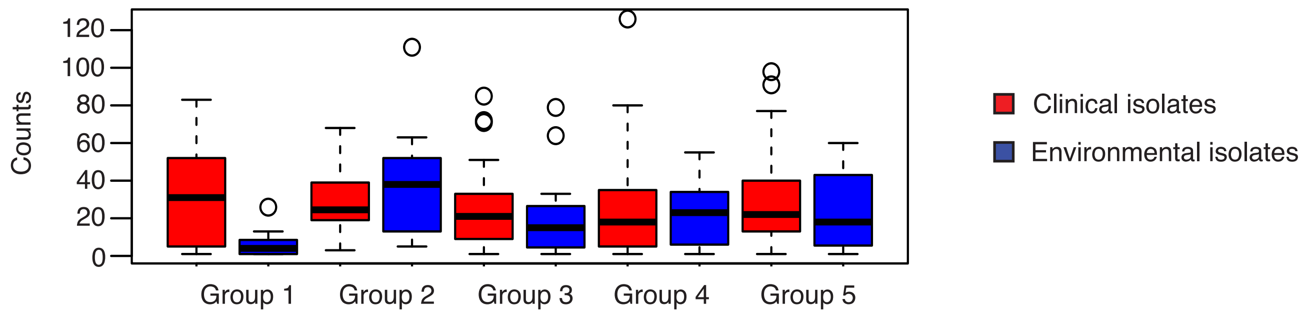

b DNA donors for recombination events detected in **clinical isolates**

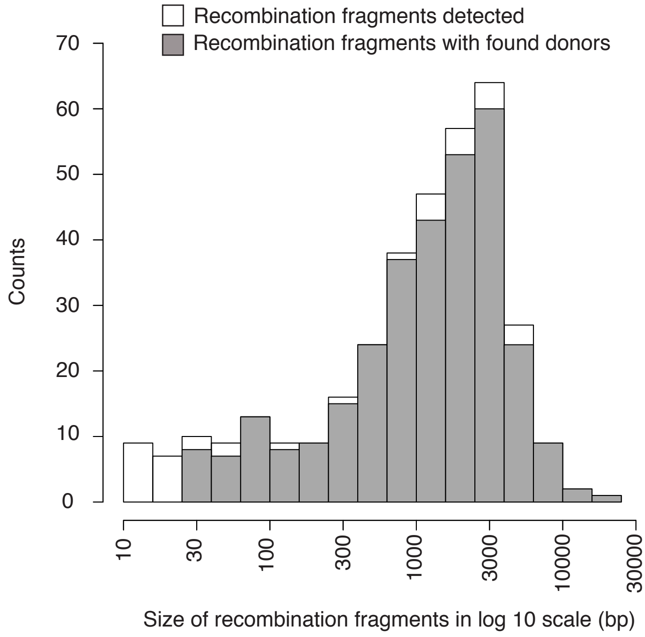

c DNA donors for recombination events detected in **environmental isolates**

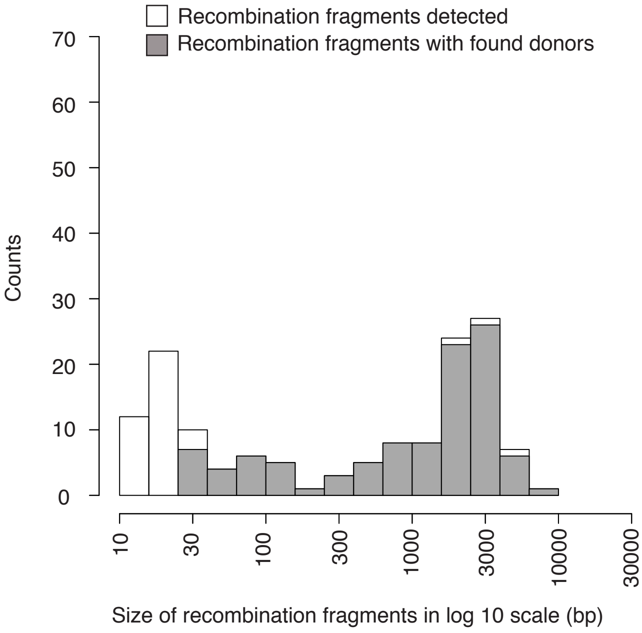

d Probability of individual isolate in being DNA donors for **clinical isolates**

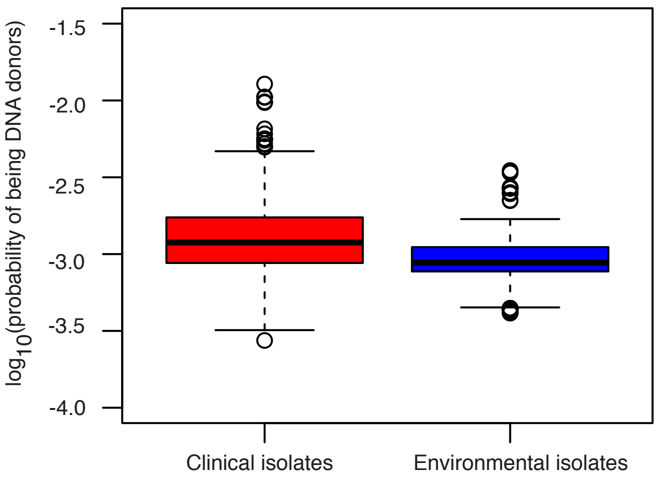

Two-sided Mann-Whitney U test p-value $<2.2 \times 10^{-16}$

e Probability of individual isolate in being DNA donors for **environmental isolates**

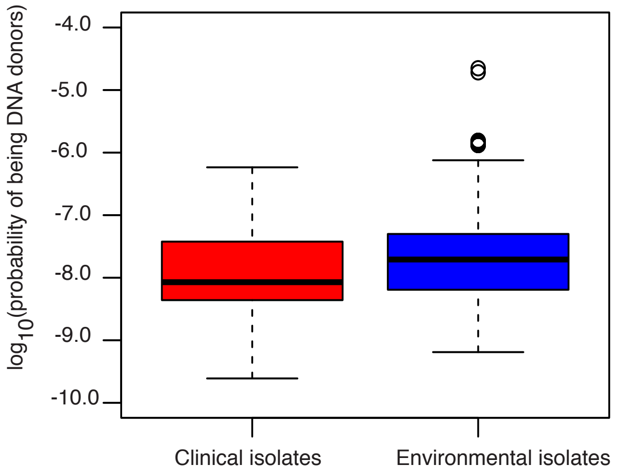

Two-sided Mann-Whitney U test p-value $= 9.41 \times 10^{-9}$

Supplementary Figure 2 Recipients and donors of recombination. (a) summarises number of recent recombination events (defined as recombination fragments detected at the tip of the phylogeny and limited to a single isolate) in the studied groups. Red and blue boxplots highlight the distribution of recent recombination in clinical and environmental isolates, respectively. (b) and (c) characterise blocks of recombination fragments detected from clinical and environmental recipients, respectively. Histograms in (b) and (c) show distribution of length of recipient blocks. Shaded in white are all recipient blocks used as queries for blast searches. In grey are recipient blocks where identical hits were detected from the rest of the population. (d) and (e) summarises the distribution of donating probability of isolates for clinical and environmental recipients, respectively. The probability is grouped by origin of donors – clinical isolate donors (red) and environmental isolate donors (blue). Two-sided Mann-Whitney U test was used to compare the preference in each recipient-donor category.

Supplementary Figure 3

a

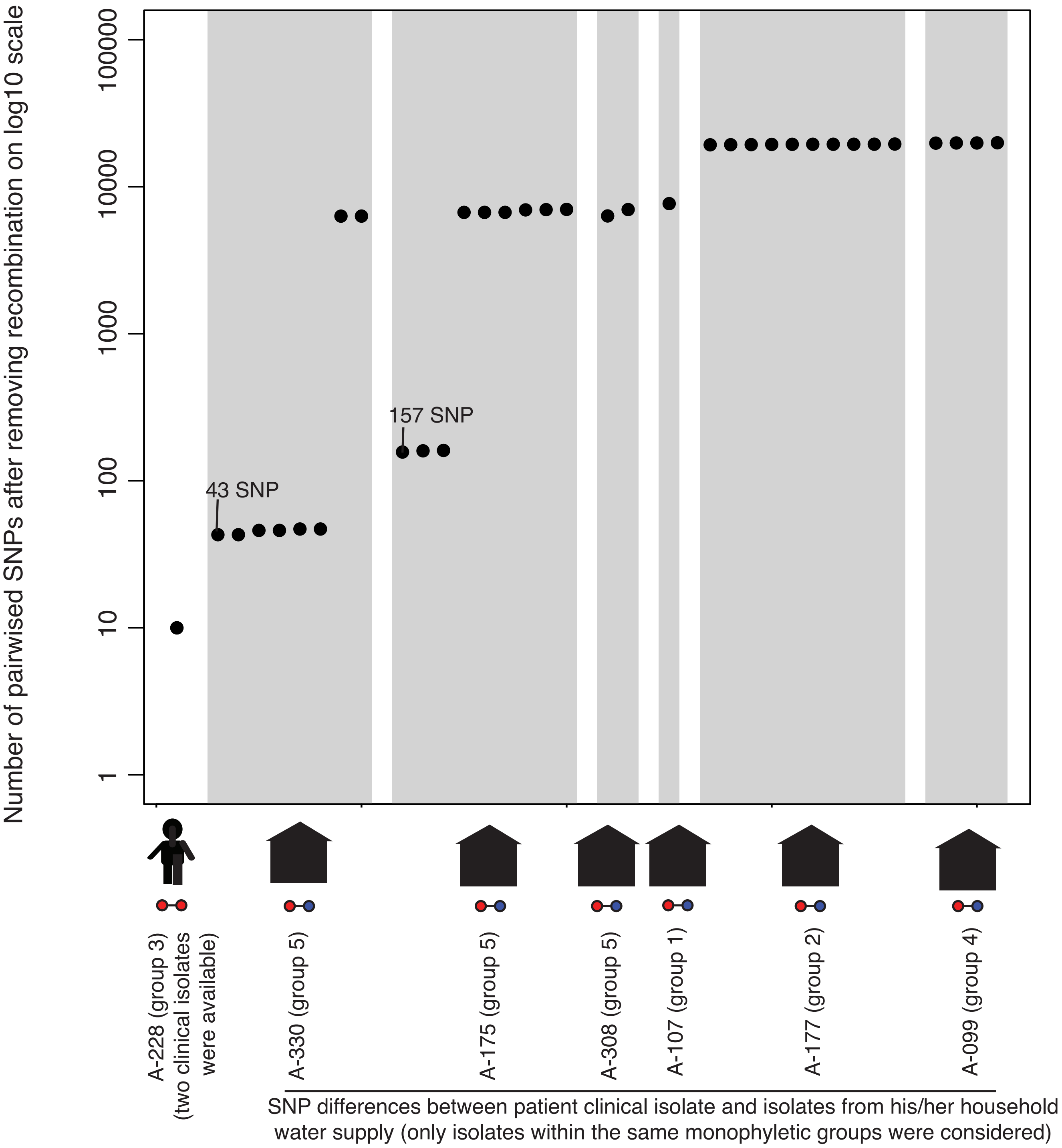

b

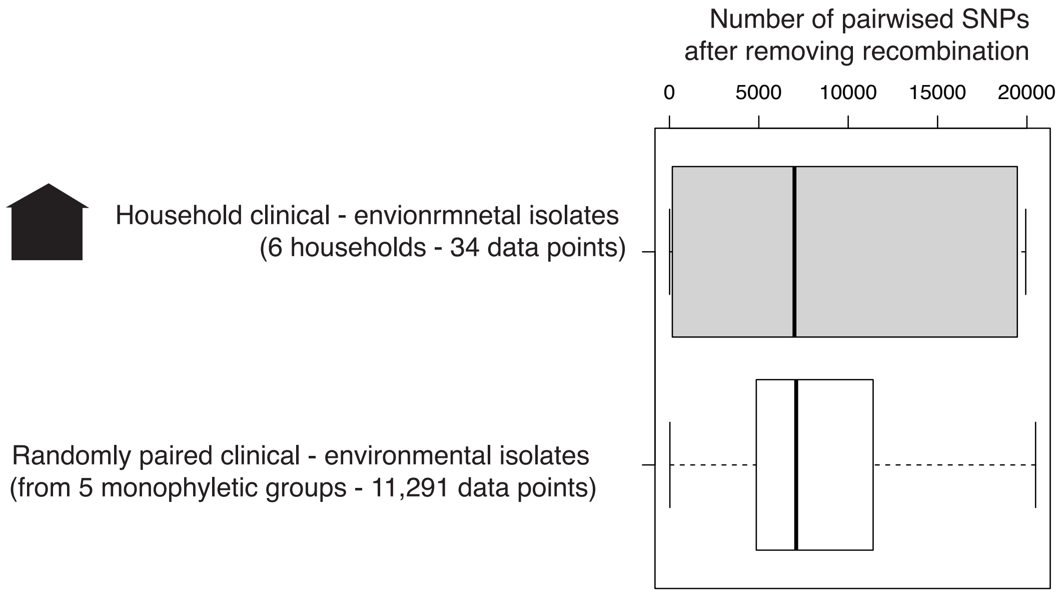

Supplementary Figure 3 Epidemiological analyses of clinical and all environmental isolates collected from each patient and his or her household. a) present 6 cases of melioidosis where both clinical and environmental isolates were clustered in the same monophyletic group. The pairwise SNP distance of two clinical isolates cultured from the same patient was plotted as a threshold (10 SNPs). Signals from recombination were removed from the analysis. (b) Boxplots compare the pairwise SNP distance between clinical and environmental isolates cultured from the same household (grey); and randomly paired clinical and environmental isolates from elsewhere (white). Two-sided Mann-Whitney U test was used to compare the genetic distance within household sampling vs randomly paired samples

Supplementary Figure 4

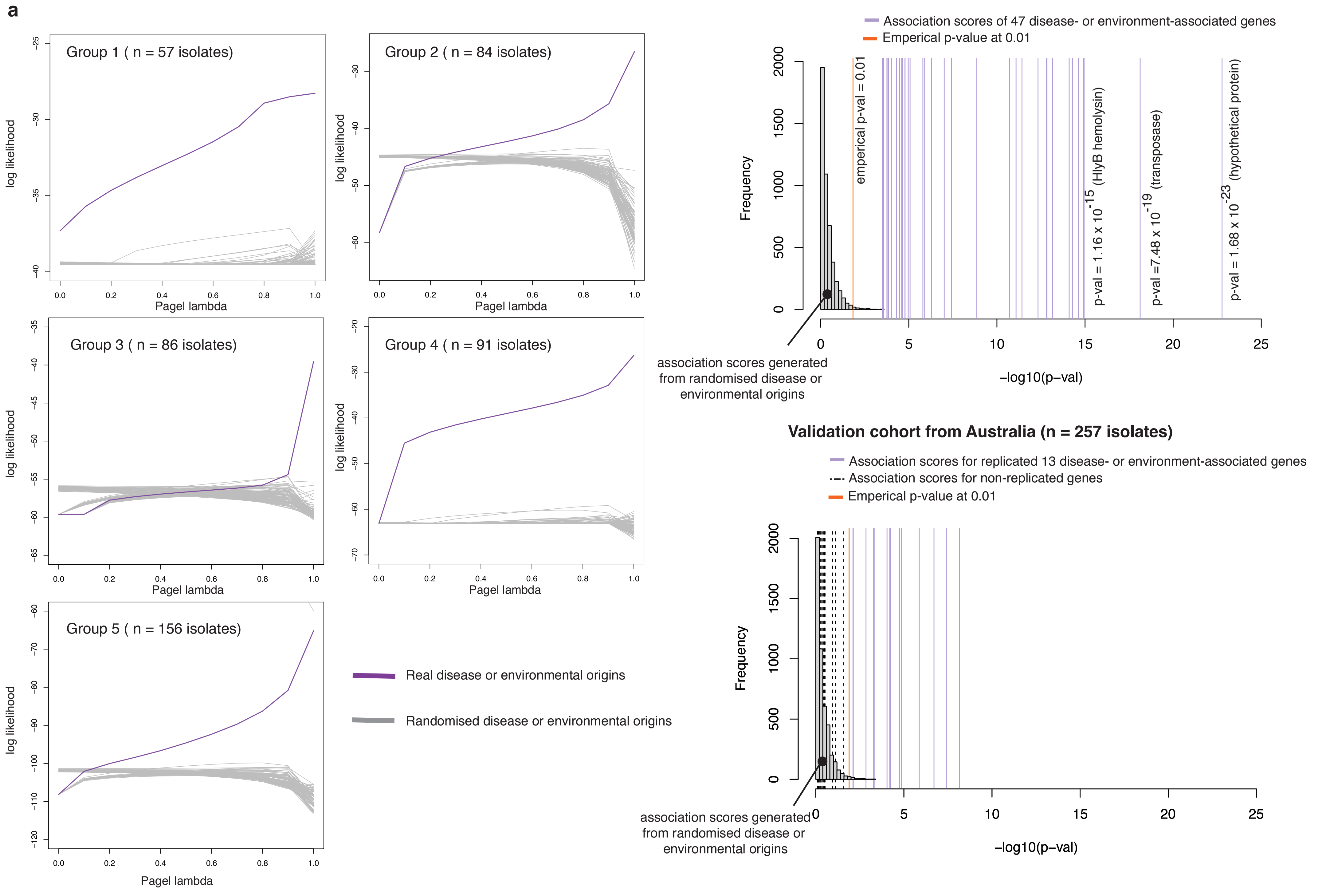

Supplementary Figure 4 Tests for true and randomized signals (a) Measurement of phylogenetic signals correlated with bacterial clinical and environmental origins. Each panel summarises a distribution of Pagel's obtained from data with true bacterial origin (purple) vs 100 permuted data with randomised bacterial origins (grey). The plots represent results from individual monophyletic groups. Log-likelihood (y-axis) was used to estimate the fitness of source following tree transformation by Pagel's λ (x-axis). A multiplication to internal branches with $\lambda = 0$ created a basal tree, while a multiplication with $\lambda = 1$ kept the tree unchanged. (b) Randomisation were performed for both discovery (top) and validation (bottom) datasets to determine an empirical p-value cut-off for the analyses. Histograms display scores generated from randomized associations. For each tested gene, 100 permutations with true genotypes but randomized source of isolate (clinical or environmental) was performed. P-values from true associations were plotted as vertical lines. Candidate genes from discovery and validation dataset that achieved significant association at p-value < 0.01 with Benjamini-Hochberg correction were also significant at an empirical p-value < 0.01 (purple lines).

Supplementary Figure 5

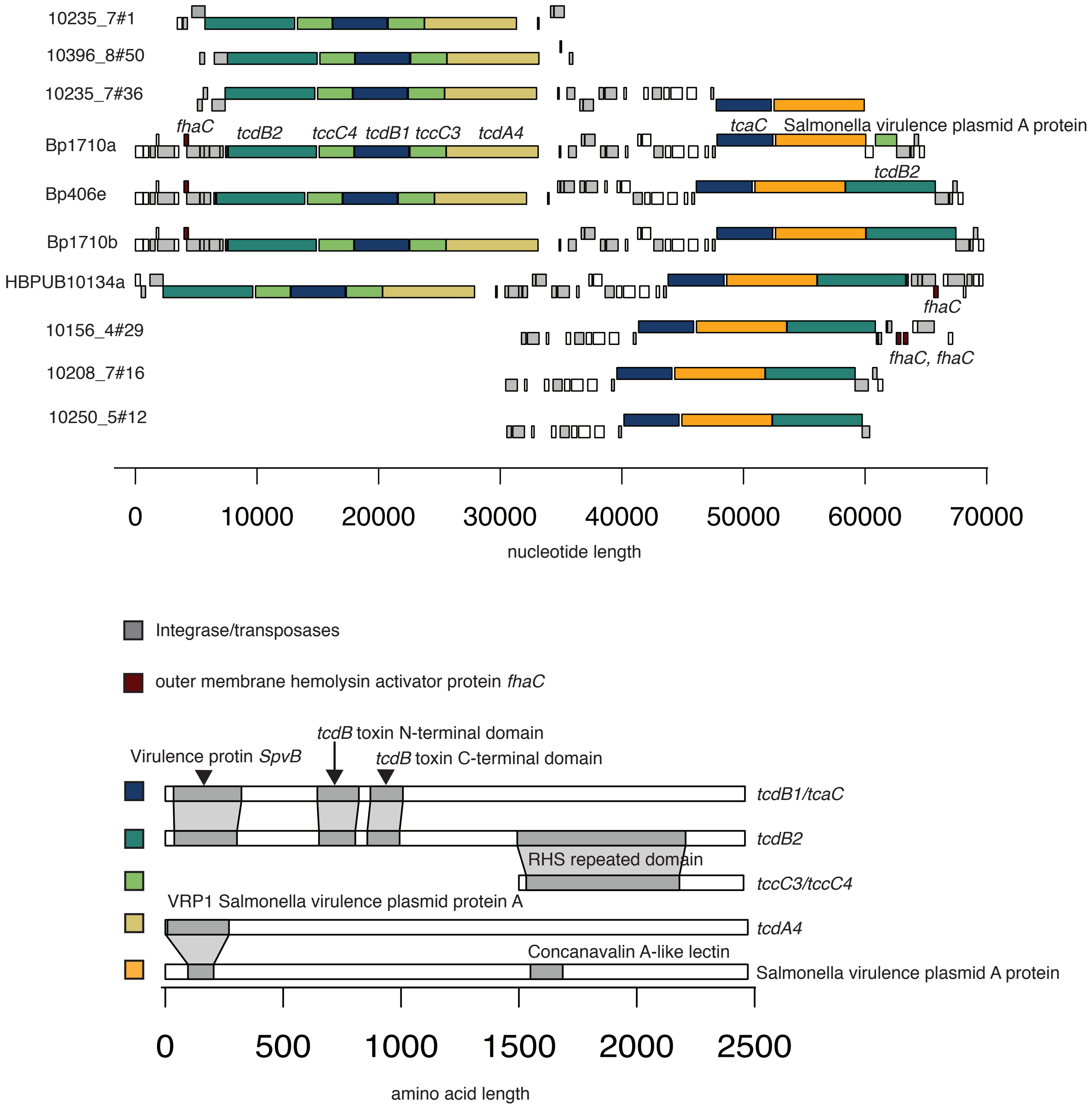

Supplementary Figure 5 The genetic architecture of toxin-complexes, dominant hits comprising toxin genes that have been characterised previously in other bacterial species and transposable elements. Gene order was obtained from reference and newly assembled genomes from the discovery cohort. Integrase/transposases spanning the complexes were highlighted in grey. Architecture of protein domains in *tcdB1*, *tcdB2*, *tccC3*, *tcdA4* and Salmonella virulence plasmid A protein gene were also annotated.

Supplementary Figure 6

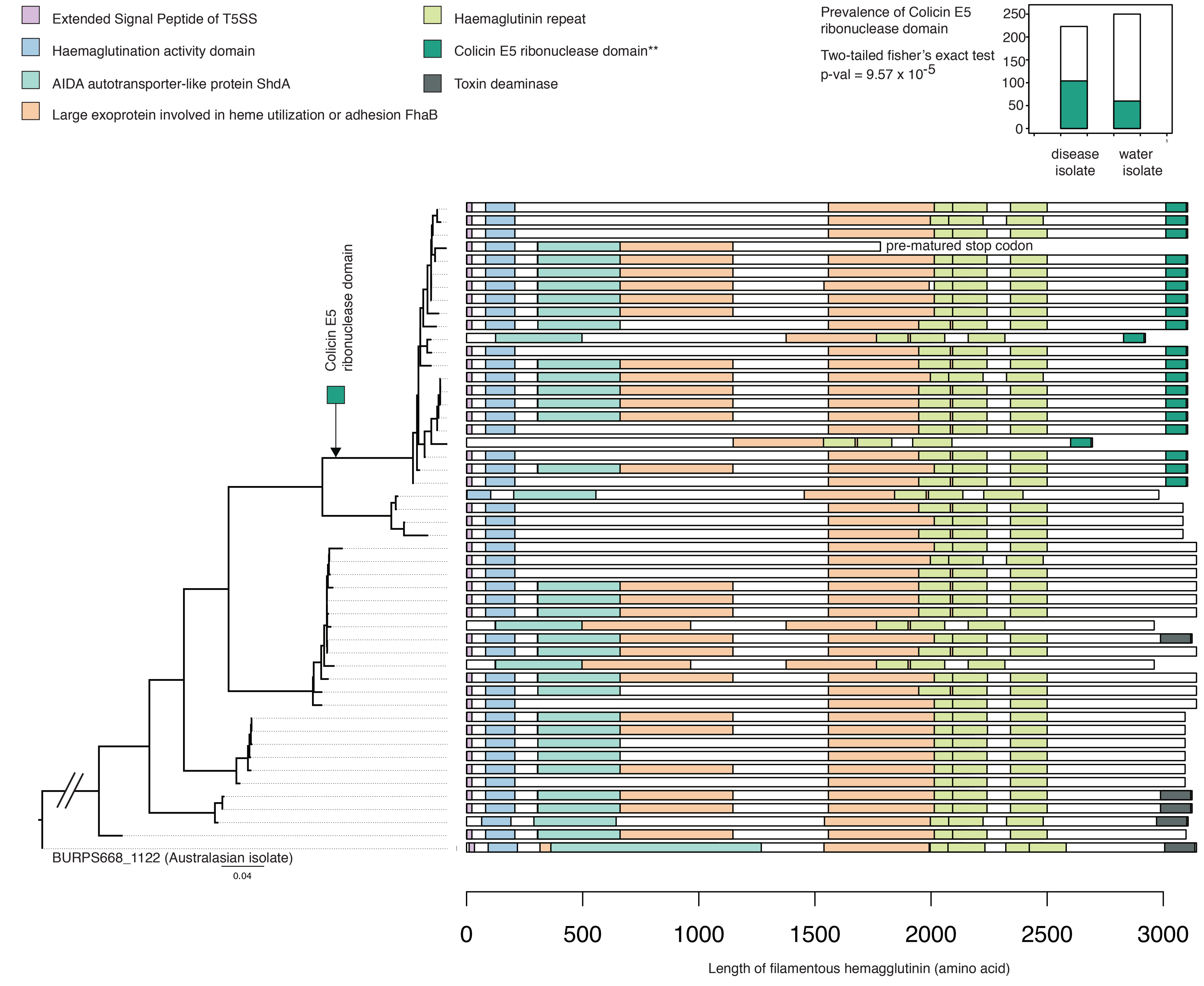

Supplementary Figure 6 Architecture of protein domains of the filamentous hemagglutinin (*BPSS2053*), and the truncated form associated with the environmental isolates. A maximum likelihood phylogenetic tree (left) was constructed from 49 *fhaB* alleles found in the discovery dataset rooted on *fhaB* from an Australian outgroup (Bp668). Protein domains (right) were annotated onto each allele, highlighting the truncated form of filamentous hemagglutinin. The diagram also highlights loss of toxin deaminase, and gain of Colicin E5 ribonuclease domain at the C-terminus, respectively. The latter was present at an elevated frequency in clinical versus environmental isolates.
