# Supplementary material for: Genetic variation associated with infection and the environment in the accidental pathogen Burkholderia pseudomallei
Source: Commun Biol. 2019 Nov 22;2:428. doi: 10.1038/s42003-019-0678-x (PMC6874650; doi:10.1038/s42003-019-0678-x)
Supplement: Supplementary file 2 — Description of Additional Supplementary Files [file 42003_2019_678_MOESM2_ESM.docx]

**Description of Supplementary Data Files**

**Supplementary data 1** Epidemiological data for discovery cohort from Northeast Thailand. Isolate and accession codes for both short reads, assemblies and annotations associated with data deposited in the European Nucleotide Archive (ENA). Environmental-disease category, date of collection, Global Positioning System (GPS) data (where available) and inferred multi-locus sequence typing (MLST) are also shown.

**Supplementary data 2** Epidemiological data for the validation cohort from Australia. Isolate and accession codes for short reads or assemblies deposited in NCBI data, as well as spatial and temporal information associated with each isolate were tabulated.

**Supplementary data 3** Disease- and environment-associated genes co-detected by two independent genome-wide association approaches. Annotations, locus information, functional category, allele frequency, evolutionary trajectory including dN/dS and number of times the genes had been acquired or lost were tabulated.

**Supplementary data 4** Presence and absence of genes in the discovery collection from Thailand

**Supplementary data 5** Presence and absence of genes in the validation cohort from Australia

**Supplementary data 6** Functional category defined by COGs, literatures, and GO terms associated with disease- and environment-associated genes. Functional enrichment analysis was performed by comparing frequency of these terms in disease- and environment-associated genes against their distributions in reference genomes.

**Supplementary data 7** Pan-genome analysis listing all genes from taxa in the discovery dataset (753 isolates) and two references (K96243 and an Australasian outgroup Bp668). Due to the file size, this has been archived in <https://figshare.com/articles/Supplementary_Data_7/10006766>

(doi: [10.6084/m9.figshare.10006766](https://doi.org/10.6084/m9.figshare.10006766))

**Supplementary data 8** Kmer-based genome-wide association analysis listing all kmers from searches for enrichment in disease and environmental isolates and the frequency in the population. The first three columns summarise the kmer frequency in the population, the raw counts of each kmer in disease isolates and environmental isolates, respectively. The fourth column represent kmer sequence, while the fifth to twelfth columns summarise association statistics

**Supplementary data 9** Gene-based genome-wide association analysis listing all genes from two reciprocal searches for enrichment in disease and environmental isolates.

**Supplementary data 10** Sequence of disease- and environmental associated genes.

Due to large file size, individual gene sequences are archived in <https://figshare.com/articles/Supplementary_Data_10/10006829>

(doi: [10.6084/m9.figshare.10006829](https://doi.org/10.6084/m9.figshare.10006829))

**Supplementary data 11** Source data used to plot Figure 2a

**Supplementary data 12** Source data used to plot Figure 3a

**Supplementary data 13** Source data used to plot Figure 3b

**Supplementary data 14** Source data used to plot Figure 5a

**Supplementary data 15** Source data used to plot Figure 5b
