# Supplementary material for: Genetic variation associated with infection and the environment in the accidental pathogen Burkholderia pseudomallei
Source: Commun Biol. 2019 Nov 22;2:428. doi: 10.1038/s42003-019-0678-x (PMC6874650; doi:10.1038/s42003-019-0678-x)
Supplement: Supplementary file 18 — Reporting Summary [file 42003_2019_678_MOESM18_ESM.pdf]

nature research

Corresponding author(s): Chewapreecha and Peacock

Last updated by author(s): Oct 20, 2019

Reporting Summary

Nature Research wishes to improve the reproducibility of the work that we publish. This form provides structure for consistency and transparency in reporting. For further information on Nature Research policies, see Authors & Referees and the Editorial Policy Checklist.

Statistics

For all statistical analyses, confirm that the following items are present in the figure legend, table legend, main text, or Methods section.

n/a	Confirmed	
☐	☒	The exact sample size (*n*) for each experimental group/condition, given as a discrete number and unit of measurement
☐	☒	A statement on whether measurements were taken from distinct samples or whether the same sample was measured repeatedly
☐	☒	The statistical test(s) used AND whether they are one- or two-sided<br>*Only common tests should be described solely by name; describe more complex techniques in the Methods section.*
☐	☒	A description of all covariates tested
☐	☒	A description of any assumptions or corrections, such as tests of normality and adjustment for multiple comparisons
☐	☒	A full description of the statistical parameters including central tendency (e.g. means) or other basic estimates (e.g. regression coefficient) AND variation (e.g. standard deviation) or associated estimates of uncertainty (e.g. confidence intervals)
☐	☒	For null hypothesis testing, the test statistic (e.g. *F*, *t*, *r*) with confidence intervals, effect sizes, degrees of freedom and *P* value noted<br>*Give P values as exact values whenever suitable.*
☒	☐	For Bayesian analysis, information on the choice of priors and Markov chain Monte Carlo settings
☒	☐	For hierarchical and complex designs, identification of the appropriate level for tests and full reporting of outcomes
☒	☐	Estimates of effect sizes (e.g. Cohen's *d*, Pearson's *r*), indicating how they were calculated

Our web collection on statistics for biologists contains articles on many of the points above.

Software and code

Policy information about availability of computer code

Data collection	No software was used to collect the data
Data analysis	All tools and R packages used for the analysis are publicly available and fully described in the method sections and noted in references (refs 34-35, 47-49, 51-57, 60-64 and 71-73)

For manuscripts utilizing custom algorithms or software that are central to the research but not yet described in published literature, software must be made available to editors/reviewers. We strongly encourage code deposition in a community repository (e.g. GitHub). See the Nature Research guidelines for submitting code & software for further information.

Data

Policy information about availability of data

All manuscripts must include a data availability statement. This statement should provide the following information, where applicable:

- Accession codes, unique identifiers, or web links for publicly available datasets
- A list of figures that have associated raw data
- A description of any restrictions on data availability

Short reads for isolates are archived in ENA or NCBI database. Accession number for each individual isolate in discovery and validation dataset are given in Supplementary data 1 and 2, respectively. Raw results from pan-genome analysis, kmer-based genome-wide associated analysis, and gene-based genome-wide association analysis are provided as Supplementary Data 7, 8 and 9, respectively.

Field-specific reporting

Please select the one below that is the best fit for your research. If you are not sure, read the appropriate sections before making your selection.

☐ Life sciences ☐ Behavioural & social sciences ☒ Ecological, evolutionary & environmental sciences

For a reference copy of the document with all sections, see nature.com/documents/nr-reporting-summary-flat.pdf

Ecological, evolutionary & environmental sciences study design

All studies must disclose on these points even when the disclosure is negative.

Study description	Our study identified Burkholderia pseudomallei gene clusters that distinguish between isolates associated with human disease and the environment. We tested genetic connection between clinical isolates collected from the patients and environmental isolates collected from patients' drinking water as well as their household water supplies, indicating that not all exposure had led to disease. Using two independent methods, we performed genome-wide association study to identify genetic determinants in a dataset from Thailand and validated our findings in a dataset from Australia. We next measured the frequency at which these genetic markers were gained or lost throughout the bacterial evolutionary history, reflecting the bacterial adaptability to a wide range of ecological niches.
Research sample	Two bacterial collections were used for discovery and validation datasets. The discovery cohort was sought from 325 clinical and 428 environmental isolates cultured from a circumscribed area of Northeast Thailand (total 753 isolates). The validation cohort was sought from 184 clinical and 73 environmental isolates from Australia (total 257 isolates, refs 18-23). Temporal, spatial and categorical structure of the samples are summarised in Figure 1.
Sampling strategy	The discovery and validations were chosen to represent two independent and distinct regions where melioidosis is highly endemic.
Data collection	For discovery dataset, the data collection was part of the study conducted in ref. 5. For validation dataset, the analysis is based on available data in the public domain (refs 18-23).
Timing and spatial scale	Timing and spatial scale of both discovery and validation dataset are limited by the availability of data from previous studies (refs 5, and 18-23).
Data exclusions	Sequences were excluded when contamination were suspected. Taxonomic identity was assigned to control for sample mix-up from other species.
Reproducibility	Independent validation dataset was used to replicate finding in discovery dataset.
Randomization	The group allocation was based on bacterial phenotype (clinical or environmental origin). However, in the test for potential genetic signal, the phenotype was randomised (100 permutations, Supplementary Figure 5) to show that the signal from the actual phenotype is not random.
Blinding	Researcher performing genomic analyses was not blinded to the data category.

Did the study involve field work? ☐ Yes ☒ No

Reporting for specific materials, systems and methods

We require information from authors about some types of materials, experimental systems and methods used in many studies. Here, indicate whether each material, system or method listed is relevant to your study. If you are not sure if a list item applies to your research, read the appropriate section before selecting a response.

Materials & experimental systems

n/a	Involved in the study	
☒	☐	Antibodies
☒	☐	Eukaryotic cell lines
☒	☐	Palaeontology
☒	☐	Animals and other organisms
☒	☐	Human research participants
☒	☐	Clinical data

Methods

n/a	Involved in the study	
☒	☐	ChIP-seq
☒	☐	Flow cytometry
☒	☐	MRI-based neuroimaging
