# Supplementary material for: Genetic variation associated with infection and the environment in the accidental pathogen Burkholderia pseudomallei
Source: Commun Biol. 2019 Nov 22;2:428. doi: 10.1038/s42003-019-0678-x (PMC6874650; doi:10.1038/s42003-019-0678-x)
Supplement: Supplementary file 19 — Peer Review File [file 42003_2019_678_MOESM19_ESM.pdf]

Reviewers' comments:

Reviewer #1 (Remarks to the Author):

This paper attempts to identify genetic signatures associated with either a clinical or environmental origin in B. pseudomallei. Unlike many bacterial GWAS studies, this study uses a large set of genomes where questions like these can begin to be asked. The conclusion of the study is that 47 genomic regions appear to be associated with either clinical or environmental strains. After reading the manuscript a couple of times, I was still unsure of how conserved the regions were in either group. Table S4 clearly shows the prevalence of genes in the Australian set, but I'm not sure about in the Thai strains; adding this information to Table S3 would be helpful. I think, in general, using even a FDR-adjusted p-value may not be biologically meaningful considering the huge number of genes present in the B. pseduomallei pan-genome. A better approach may be a Monte Carlo analysis to identify the number of regions that are associated by mere chance. When I went to investigate the associated genes, I couldn't correlate the IDs with anything meaningful: using locus tags would help the reader track down the genes of interest. And finally, I found the methods on the Kmer analysis to be a little too vague for full reproducibility. Additional specific comments/questions:

25: This is an estimate based on a model and not real death data. This needs to be clarified as it is currently misleading
L130: I don't understand the argument for not introducing false positive associations. This needs additional clarification.
L131: What is the frequency of presence/absence of these 38,813 Kmers across the different groups? Perhaps add this as a column to one of the supplemental data files?
L138: I"m unsure of how you grouped the 47 genes into 26 loci. Was this dones using gene synteny or just "size"? The methods don't make this clear.
L139: How universally conserved are these Kmers?
L144: It's unclear of whether these are deletions or SNVs that create different Kmers
L250: There are only 257 genomes in Table S2, not 258
L286: How were Kmers created and what size? Were reverse complement Kmers included? This seems to be critical part of the study and more detail is needed
L314: I'm unsure what you mean by rooting on a single gene homologue. Please clarify.
L317: What is Pagel's lambda? Some background and motivation would be appreciated.
L331: It is unclear of how you generated Kmers. You mean that every genome was split into Kmers of 9-100 bases, overlapping by 1?
L334: Why would you not include Kmers that occurred in >95% of samples?
L346: Fix "to allowed for"
L357: Citation for Benjamini-Hochberg correction?

Reviewer #2 (Remarks to the Author):

This detailed genomic comparison of Burkholderia strains, both environmentally and clinically isolated, shows the enormous power of large amounts of high quality NGS data coupled with robust statistics, especially when placed in context using sound information regarding the microbe. I found the manuscript well written and highly informative. Any potential issues with cross categorization are noted and their impact on the results should be negligible.

Reviewer #3 (Remarks to the Author):

The authors present a genomic and GWAS analysis of Burkolderia pseudomallei clinical and environmental isolates. They use a large discovery cohort (Thailand) and validation cohort to identify candidate genomic regions associated to disease in humans.

Overall, the manuscript is well written, the GWAS approach is very interesting and the conclusions are sound. I just have some comments I would like the authors to address:
1. The lack of a clear link between clinical and environmental isolates from the same household is clearly shown. However I think the authors can try to link clinical isolates to other sources in their dataset. Inspecting the phylogeny some clinical isolates cluster together with other environmental/households. Is it the case? Can you report the closest isolate to each clinical isolate in the dataset, the distance and whether it is environmental or clinical?
2. Clinical isolates also tend to cluster together in the phylogeny, part of it will be a bias effect because of the sampling scheme but, are there clinical samples close enough to suggests a common source of infection?
3. In the same lines, I don't know how much data the authors have for this but instead of limiting analyses to the same household, is it not possible to expand the analysis to wider geographic locations? It is clear from your analysis that many cases have been infected elsewhere and maybe there are common sources? Also if you have geo-positioning data, is there a correlation between genetic distance between isolates and geographic distance which can suggests common sources between isolates?
4. A general concern is how well culture represents diversity and particularly if there are differences in culture recovery rates of human vs environmental. I know this is difficult to estimate but I would like a more extended discussion on this for scientist not familiarized with Burkholderia.
5. Page 94. When accounting for genetic distances between household pairs you mention that the closest is A-175 and the isolates are still 483 SNPs apart. This corresponds to around 130 years. I am not familiarized with Burkholderia, is it standard practice not to account for recombination when doing these analyses? In line 93 you "assume" a constant rate of mutation and recombination. However does it not makes more sense to detect and remove recombinant tracks and then calculate distances and timing?
6. In general there are several reports showing the recombinogenic nature of B pseudomallei but no effort is done to detect and account for recombination? How much recombination is between isolates? How much recombination contributes to the overall diversity? Is recombination linked to the candidate genes later in the manuscript?
7. In fact, you don't find a direct link between isolates in the household clinical-enviromental interface. However recombination can give you and idea on the likelihood that your clinical strains have in fact share a niche with environmental isolates? I think establishing those recombination links can be helpful in this case
8. In the same lines, phylogenies should be reconstructed without recombination tracks (if any)
9. Line 162 – can you report in the main text the dN/dS values for the statistical comparison, it will be easier to follow. Also can you calculate a dN/dS for the non-core genes, at least for those with a minimal number of isolates? It will be interesting to see how much the nine genes depart from the rest of the genome as the core is likely enriched by essential genes
10. Minor comment. Line 20 – abstract. I think that it is better to say "likely implicated".

15th September 2019

We are immensely grateful for reviewers for constructive and helpful comments, which has led to a much improved manuscript. We have aimed to address these points in full. Changes in the manuscript text file are highlighted in yellow. Please see below.

Reviewers' comments:

Reviewer #1 (Remarks to the Author):

This paper attempts to identify genetic signatures associated with either a clinical or environmental origin in *B. pseudomallei*. Unlike many bacterial GWAS studies, this study uses a large set of genomes where questions like these can begin to be asked. The conclusion of the study is that 47 genomic regions appear to be associated with either clinical or environmental strains.

After reading the manuscript a couple of times, I was still unsure of how conserved the regions were in either group. Table S4 clearly shows the prevalence of genes in the Australian set, but I'm not sure about in the Thai strains; adding this information to Table S3 would be helpful.

We thank the referee for the suggestion. Additional information on the conservation of both genes in the Thai and Australian strains have been added in our new ***Supplementary table 4*** *and* ***5****, respectively.*

I think, in general, using even a FDR-adjusted p-value may not be biologically meaningful considering the huge number of genes present in the *B. pseduomallei* pan-genome. A better approach may be a Monte Carlo analysis to identify the number of regions that are associated by mere chance.

We are happy to provide the additional analysis suggested by the reviewer. Randomization tests were performed for genes in both discovery and validation datasets to determine an empirical p-value for the cut-off threshold. For each gene, 100 permutations were run with true genotypes (gene presence or absence) but randomized source of isolate (clinical or environment). This process generated randomized probability distributions outlined in our new ***Supplementary Figure 5b****. Our new results showed that candidate genes achieving significant associations at p-value <0.01 with Benjamini-Hochberg correction also achieved significant associations with an empirical p-value < 0.01, suggesting that the associations were not random. Description of the Monte Carlo permutation test were added to the manuscript text (line 410-419) and Supplementary Figure 5 legend*

When I went to investigate the associated genes, I couldn't correlate the IDs with anything meaningful: using locus tags would help the reader track down the genes of interest. And finally, I found the methods on the Kmer analysis to be a little too vague for full reproducibility.

We thank the referee for this suggestion. We have linked the IDs with the locus tags on the reference genome K96243 where they are available. However, for genes absent from the reference genome, IDs are still used. The annotation of the locus tags and IDs are provided in ***Supplementary table 3****, with full gene annotations labeled in* ***Figure 3*** *&* ***4*** *to facilitate*

*biological interpretation. Moreover, full sequences of these locus tags and IDs are provided to allow reproducibility of the data (**Supplementary data 4**). We also expanded description and the syntax used for Kmer analysis session to ensure that the method is clearly communicated and reproducible (lines 358-384).*

Additional specific comments/questions:

25: This is an estimate based on a model and not real death data. This needs to be clarified as it is currently misleading

We apologize for a misleading statement. This has been rectified on line 23.

L130: I don't understand the argument for not introducing false positive associations. This needs additional clarification.

The new statement is expanded as "We noted that there was potential cross categorization as the environmental isolates could be capable of causing the disease. While this caveat reduces the power to detect association which elevates the true negatives, this would be unlikely to impact on the false positive rate."(line 128-131)

L131: What is the frequency of presence/absence of these 38,813 Kmers across the different groups? Perhaps add this as a column to one of the supplemental data files?

*We thank the referee for this suggestion. We have expanded this as columns in the **supplementary data 2***

L138: I"m unsure of how you grouped the 47 genes into 26 loci. Was this dones using gene synteny or just "size"? The methods don't make this clear.

The group was based on the "size" of transcription fragments (Ooi et al 2013). The text has been rewritten to clarify this on lines 138-139

L139: How universally conserved are these Kmers?
*The conservation of kmers across the population is additionally tabulated in **Supplementary data 2**.*

L144: It's unclear of whether these are deletions or SNVs that create different Kmers
This detail has been expanded in the method section in line 382-384.

L250: There are only 257 genomes in Table S2, not 258

We apologize for this mistake. This has been corrected.

L286: How were Kmers created and what size? Were reverse complement Kmers included? This seems to be critical part of the study and more detail is needed

This detail has been expanded in line 359-380. Briefly, kmers were counted from the assemblies into 9-100 bp length of fragments using fsm-lite. Significant kmers were interpreted by mapping them back to the annotated pan-genome. The mapping was allowed for match on both forward and reverse strands.

L314: I'm unsure what you mean by rooting on a single gene homologue. Please clarify.

This refers to "single-copy" gene. We have rewritten relevant method section to clarify this (line 290, and 303).

L317: What is Pagel's lambda? Some background and motivation would be appreciated.

This detail has been expanded in line 346-348.

L331: It is unclear of how you generated Kmers. You mean that every genome was split into Kmers of 9-100 bases, overlapping by 1?

This detail has been expanded in line 359-380.

L334: Why would you not include Kmers that occurred in >95% of samples?

Kmers occurred in >95% samples are commonly shared in both environmental and clinical isolates, and unlikely distinguish environmental or clinical subgroups. We thus exclude these kmers to reduce computational loads.

L346: Fix "to allowed for"

Corrected as suggested

L357: Citation for Benjamini-Hochberg correction?

Corrected as suggested (new ref. 65)

Reviewer #2 (Remarks to the Author):

This detailed genomic comparison of *Burkholderia* strains, both environmentally and clinically isolated, shows the enormous power of large amounts of high quality NGS data coupled with robust statistics, especially when placed in context using sound information regarding the microbe. I found the manuscript well written and highly informative. Any potential issues with cross categorization are noted and their impact on the results should be negligible.

We thank the reviewer for their positive comments.

Reviewer #3 (Remarks to the Author):

The authors present a genomic and GWAS analysis of *Burkolderia pseudomallei* clinical and environmental isolates. They use a large discovery cohort (Thailand) and validation cohort to identify candidate genomic regions associated to disease in humans.

Overall, the manuscript is well written, the GWAS approach is very interesting and the conclusions are sound. I just have some comments I would like the authors to address:

1. The lack of a clear link between clinical and environmental isolates from the same household is clearly shown. However, I think the authors can try to link clinical isolates to other sources in their dataset. Inspecting the phylogeny some clinical isolates cluster together with other environmental/households. Is it the case? Can you report the closest isolate to each clinical isolate in the dataset, the distance and whether it is environmental or clinical?

In light of the reviewer's comment, we have now also investigated other plausible sources of infection. This investigation is expanded in the new result section (line 89-116) and new ***Figure 2****. We tested for the possibility of water supply from different households being the source of infection by pooling all water isolates together, and identifying the closest environmental isolate that could be the source of infection for each clinical isolate. To ensure greater confidence of these identifications, these analyses were limited to 5 where we could confidently detect recombination as the reliable detection of recombination is known to decrease with distantly related lineages.*

2. Clinical isolates also tend to cluster together in the phylogeny, part of it will be a bias effect because of the sampling scheme but, are there clinical samples close enough to suggests a common source of infection?

We thank the referee and went to investigate the potential common source of infection as suggested. ***Figure 2c – 2g*** *highlighted spatial location where samples were collected and their genetic links. However, these isolates appeared to disperse through entire geographical locations. Therefore, the common source of infection could not be confidently defined.*

3. In the same lines, I don't know how much data the authors have for this but instead of limiting analyses to the same household, is it not possible to expand the analysis to wider geographic locations? It is clear from your analysis that many cases have been infected elsewhere and maybe there are common sources? Also if you have geo-positioning data, is

there a correlation between genetic distance between isolates and geographic distance which can suggests common sources between isolates?

In light of referee comment, we expanded the analysis to wider geographical location and investigated the correlation between the genetic and spatial distance of each clinical and its genetically closest environmental isolate (new ***Figure 2b****, line 102-116). However, there was a lack of correlation. It is possible that the Mun river, its canal systems and floodplains might have dispersed isolates that share the same recent common ancestors over a long distance, thereby destroying spatial signals for common source of infection. This discussion was added to the result section.*

4. A general concern is how well culture represents diversity and particularly if there are differences in culture recovery rates of human vs environmental. I know this is difficult to estimate but I would like a more extended discussion on this for scientist not familiarized with Burkholderia.

We have noted a difference in recovery rate of B. pseudomallei cultured from water samples (1 x 10^{-3} *CFU/ml) and clinical samples (blood bottle: 1 CFU/ml) in line 241 -243.*

5. Page 94. When accounting for genetic distances between household pairs you mention that the closest is A-175 and the isolates are still 483 SNPs apart. This corresponds to around 130 years. I am not familiarized with *Burkholderia*, is it standard practice not to account for recombination when doing these analyses? In line 93 you “assume” a constant rate of mutation and recombination. However does it not makes more sense to detect and remove recombinant tracks and then calculate distances and timing?

Unfortunately, recombination cannot be reliably removed across the entire Burkholderia phylogeny but rather in monophyletic groups where strains shared close common recent ancestors. As most clinical and household water isolates were located on different phylogenetic subgroups, our initial investigation was a broad-brush attempt to investigate all isolates by generalizing the mutation and recombination rates across all backgrounds. Following the reviewer’s suggestion, we have conducted finer resolution analyses with recombination removed to investigate this fully. The results are reported in line 96-101 and ***Supplementary Figure 4****.*

6. In general there are several reports showing the recombinogenic nature of *B pseudomallei* but no effort is done to detect and account for recombination? How much recombination is between isolates? How much recombination contributes to the overall diversity? Is recombination linked to the candidate genes later in the manuscript?

In light of the above comment, we have additionally identified recombination in five monophyletic groups and compared its contribution to the overall diversity using the recombination per mutation ratio (r/m). This information and recombination were reported in ***Supplementary Figure 2****. However, disease-associated and environment-associated genes identified later in the manuscript were not all present in the K96243 reference genome where recombination detection were performed. We thus could not specifically link the candidate genes to recombination events based on K96243 reference.*

7. In fact, you don’t find a direct link between isolates in the household clinical-enviromental interface. However recombination can give you an idea on the likelihood that your clinical

strains have in fact share a niche with environmental isolates? I think establishing those recombination links can be helpful in this case.

We are grateful for the comment and went to investigate the recipient-donor relationship of the clinical and environmental isolates. We found that clinical isolates could be potential DNA donors for environmental isolates and vice versa, supporting the idea that both clinical and environmental isolates share the same niche. This analysis was added as a new section (line 69-86)

8. In the same lines, phylogenies should be reconstructed without recombination tracks (if any)

We thank the referee for comments and have reconstructed the phylogenies with recombination removed.

9. Line 162 – can you report in the main text the dN/dS values for the statistical comparison, it will be easier to follow. Also can you calculate a dN/dS for the non-core genes, at least for those with a minimal number of isolates? It will be interesting to see how much the nine genes depart from the rest of the genome as the core is likely enriched by essential genes

We have calculated the d N/ d S of accessory genes as suggested. The distribution of randomly selected accessory genes is plotted in ***Figure 4a.***

10. Minor comment. Line 20 – abstract. I think that it is better to say “likely implicated”.

This has been corrected as suggested.

REVIEWERS' COMMENTS:

Reviewer #1 (Remarks to the Author):

The authors did a good job of addressing my original concerns in this revised manuscript. There are a couple of issues that I had with some of the revisions and those are shown below. My fundamental concern is the idea that's raised in the discussion that these 47 regions can distinguish between clinical and environmental strains. I would argue that the authors only identified regions that were enriched, sometimes slightly, in each group using the isolates screened. Based on the reported frequencies of the 47 regions, I don't know how much value that they would add for studies in either diagnostics or pathogenesis. The fundamental limitation to this study, in my opinion, is that the ecology of Bp is not well studied, which is likely confounding the identification of clear clinical/environmental signatures.

L157-l158: dN/dS is not just the ratio of NS to S substitutions alone. The number of sites is critical for this calculation
L212: "Distinguish between" suggests a diagnostic capability, which I don't think seems justified.
L319-L320: What is the justification for using "recombination-free phylogenies". Did you test to see if recombination was truly removed?

Reviewer #2 (Remarks to the Author):

No additional comments

Reviewer #3 (Remarks to the Author):

The new analyses are sound and relevant. I congrat the authors for addressing all my concerns and the nice work.

We are very grateful to reviewers for their suggestions. Our point-by-point responses (in italic) are outlined below with the changes highlighted in yellow in the main text.

REVIEWERS' COMMENTS:

Reviewer #1 (Remarks to the Author):

The authors did a good job of addressing my original concerns in this revised manuscript. There are a couple of issues that I had with some of the revisions and those are shown below. My fundamental concern is the idea that's raised in the discussion that these 47 regions can distinguish between clinical and environmental strains. I would argue that the authors only identified regions that were enriched, sometimes slightly, in each group using the isolates screened. Based on the reported frequencies of the 47 regions, I don't know how much value that they would add for studies in either diagnostics or pathogenesis. The fundamental limitation to this study, in my opinion, is that the ecology of Bp is not well studied, which is likely confounding the identification of clear clinical/environmental signatures.

We thank the referee for raising this issue. Our discussion has been rewritten to address this limitation (line 272-287).

L157-l158: dN/dS is not just the ratio of NS to S substitutions alone. The number of sites is critical for this calculation

This has been corrected (line 211-213)

L212: "Distinguish between" suggests a diagnostic capability, which I don't think seems justified.

This has been changed to "enriched in [each group]" as suggested above (line 274-276).

L319-L320: What is the justification for using "recombination-free phylogenies". Did you test to see if recombination was truly removed?

This has been changed to "Phylogenies with recombination removed" (line 411)

Reviewer #2 (Remarks to the Author):

No additional comments

We thank the referee for previous positive comments.

Reviewer #3 (Remarks to the Author):

The new analyses are sound and relevant. I congrat the authors for addressing all my concerns and the nice work.

We thank the referee for positive comments.
